# Cellular basis of ABA‐induced *de novo* meristem formation and sex‐type conversion in Ceratopteris gametophytes

**DOI:** 10.1111/tpj.70543

**Published:** 2025-11-04

**Authors:** Xi Yang, Xing Liu, Yun Zhou

**Affiliations:** ^1^ Department of Botany and Plant Pathology Purdue University West Lafayette Indiana 47907 USA; ^2^ Purdue Center for Plant Biology Purdue University West Lafayette Indiana 47907 USA; ^3^ Department of Biochemistry Purdue University West Lafayette Indiana 47907 USA

**Keywords:** cell division, ABA, hermaphroditic gametophyte, male gametophyte, multicellular meristem, seed‐free plants, ferns, sex conversion, *de novo* meristem formation, Ceratopteris

## Abstract

Meristems house pluripotent stem cells and sustain continuous growth and organogenesis in land plants. Unlike seed plants, whose gametophytes lack meristems, fern gametophytes initiate and maintain meristems, enabling growth independently from sporophytes. In the model fern *Ceratopteris richardii*, gametophytes develop into either hermaphrodites or males. Hermaphrodites maintain multicellular meristems and secrete the pheromone antheridiogen, directing undetermined gametophytes to become male. Under constant antheridiogen exposure, males lack meristems and exclusively produce sperm‐bearing antheridia. The phytohormone abscisic acid (ABA) antagonizes antheridiogen signaling during this process, suggesting a crucial, lineage‐specific role of ABA in fern meristem specification. Nonetheless, the cellular dynamics underlying ABA‐induced meristem formation and sex‐type conversion remain unclear. Here, we used non‐invasive, long‐term confocal time‐lapse imaging to capture the dynamic process of ABA‐induced male‐to‐hermaphrodite conversion in Ceratopteris at single‐cell resolution. Cell lineage analyses revealed that ABA triggers the formation of a *de novo* meristem, originating entirely from a single non‐antheridium meristem progenitor cell (MPC), mirroring the meristem formation observed following antheridiogen removal. Importantly, ABA exhibited dual functions: promoting the cell fate re‐specification essential for meristem initiation and concurrently suppressing cell division within the developing meristem lineage. Genetic analyses with different combinations of antheridiogen and ABA treatments demonstrated these dual roles could be uncoupled, yet both required functional ABA signaling. Our findings reveal both conserved and lineage‐specific mechanisms for meristem initiation triggered by distinct environmental cues, providing insight into hormone‐mediated cellular reprogramming and proliferation during sex‐type specification in land plants.

## Significance Statement

This study uncovers the cellular basis of ABA‐induced *de novo* meristem formation from a single progenitor cell during sex‐type conversion in haploid fern gametophytes. These findings highlight both conserved and lineage‐specific hormone signals that regulate cell fate reprogramming and proliferation during sexual differentiation and meristem initiation in land plants.

## INTRODUCTION

Meristems perform conserved and essential functions across land plants. They house pluripotent stem cells, support continuous cell proliferation, and drive organogenesis and body formation (Harrison, 2017; Heidstra & Sabatini, 2014; Meyerowitz, 1997). In seed plants, various types of apical and lateral meristems are established during the asexual sporophyte phase, whereas their gametophytes lack meristems and rely on the sporophytes for growth (Bowman et al., 2016; Kean‐Galeno et al., 2024; Li & Ma, 2002; Mccormick, 2004). In contrast, ferns—reproducing via spores rather than seeds—initiate and maintain meristems during their gametophyte phase, enabling development independently of the sporophytes (Banks, 1999; Harrison, 2017; Imaichi, 2013; Nayar & Kaur, 1971; Plackett et al., 2015; Rensing, 2017; Rivera et al., 2018). While meristem regulation in seed plant sporophytes (e.g. Arabidopsis) has been extensively studied (Gaillochet & Lohmann, 2015; Geng et al., 2025; Han et al., 2020; Kitagawa & Jackson, 2019; Shpak & Uzair, 2025), only recently have a few studies begun to uncover the function and regulation of gametophytic meristem development in ferns (Wu et al., 2023; Wu, Yan, Liu, et al., 2022; Wu, Yan, Yang, et al., 2022; Xie et al., 2025).

The fern *Ceratopteris richardii* has been developed and widely used as a model system for studying various aspects of biological processes (Banks, 1999; Bui et al., 2015; Geng et al., 2021; Geng et al., 2024; Hickok et al., 1987; Hickok et al., 1995; Kinosian & Wolf, 2022; Marchant et al., 2022; Plackett et al., 2015; Woudenberg et al., 2024; Youngstrom et al., 2019). During the gametophyte phase, Ceratopteris develops as two distinct sex types: hermaphrodites and males (Banks, 1999; Conway & Di Stilio, 2020; Hickok et al., 1987). Hermaphroditic gametophytes establish a multicellular marginal meristem and produce both egg‐bearing archegonia and sperm‐producing antheridia. In contrast, male gametophyte development is regulated by the pheromone antheridiogen, which is synthesized and secreted by hermaphrodites (Banks, 1999; Hickok et al., 1987; Hickok et al., 1995). In the presence of antheridiogen, sexually undetermined gametophytes are directed to develop into males that lack meristems and produce only antheridia (Atallah & Banks, 2015; Hickok et al., 1987). This male developmental program is highly plastic and requires continuous exposure to antheridiogen for maintenance (Eberle & Banks, 1996; Hickok et al., 1987; Juarez & Banks, 1998). Mature males that are no longer exposed to antheridiogen can undergo sex‐type conversion, forming new meristems and archegonia, thus becoming hermaphrodites (Atallah & Banks, 2015; Cheruiyot & Schwartz, 2007; Hickok et al., 1987; Juarez & Banks, 1998). This developmental flexibility is crucial for sexual reproduction and species persistence in ferns. It allows the gametophyte population to dynamically adjust the ratio of males to hermaphrodites, preventing a scenario in which all individuals develop as males. This regulation ensures successful fertilization and promotes outcrossing to enhance genetic diversity. In a recent study, we found that the entire multicellular meristem induced by antheridiogen depletion originates from a single non‐antheridium meristem progenitor cell (MPC) within the male gametophyte (Yang et al., 2025). This MPC lineage reenters the cell cycle and maintains mitotic activity throughout the male‐to‐hermaphrodite transition (Yang et al., 2025).

Besides antheridiogen, previous studies suggest that several other hormones play roles in fern gametophyte development and sex‐type specification (Burow et al., 2025; Granados et al., 2022; Hickok, 1983; Kaźmierczak, 2007). Among them, abscisic acid (ABA) has been reported to block the antheridiogen response in Ceratopteris gametophytes, and ABA treatment promotes gametophytes to develop into hermaphrodites rather than males (Banks et al., 1993; Hickok, 1983). Antheridiogens in many ferns have been characterized as gibberellins or gibberellin‐like compounds (Furber & Mander, 1988; Näf et al., 1975; Takeno et al., 1989; Tanaka et al., 2014; Yamane et al., 1979; Yamane et al., 1987). In addition, gibberellin biosynthesis inhibitors have been shown to suppress male gametophyte development in Ceratopteris, suggesting that antheridiogen biosynthesis in Ceratopteris shares at least the early steps of the gibberellin biosynthesis pathway (Warne & Hickok, 1989). As an evolutionarily conserved antagonist of gibberellins (Liu & Hou, 2018; Shu et al., 2018; Thomas et al., 1964), ABA plays key roles in seed development and dormancy (Finch‐Savage & Leubner‐Metzger, 2006), stomata closure and leaf transpiration (Cai et al., 2017; Hsu et al., 2021; Mcadam & Brodribb, 2012; Yoshida et al., 2010), and plant stress responses (Cutler et al., 2010; Leung & Giraudat, 1998; Song et al., 2016). In Ceratopteris gametophytes, McAdam et al. identified the loss‐of‐function mutant in the homolog of OST1, a conserved ABA signaling component belonging to the SnRK2 subclade, which is insensitive to ABA treatment (Mcadam et al., 2016). In this *gaia1/ost1* mutant, ABA‐induced inhibition of spore germination is reduced, and when grown in the presence of both antheridiogen and ABA, mutant gametophytes remain male, whereas wild‐type gametophytes develop as hermaphrodites (Mcadam et al., 2016). Consistently, ABA quantification studies showed that Ceratopteris spores accumulate high levels of ABA after maturation, which remain elevated before germination and decline steadily after germination begins (Mcadam et al., 2016; Warne & Hickok, 1991). This decline in ABA likely enables antheridiogen, secreted by early‐germinated hermaphrodites, to more effectively regulate the proportion of males in the gametophyte population.

Despite these insights, the cellular basis of ABA‐induced sex determination and *de novo* meristem formation remains largely unknown. In this study, we performed long‐term time‐lapse confocal imaging to capture the dynamics of male‐to‐hermaphrodite conversion in response to ABA at high spatial and temporal resolution. Lineage and division analyses revealed that ABA robustly triggers the formation of a *de novo* multicellular meristem, which consistently originates from a single non‐antheridium cell, the meristem progenitor cell (MPC). This finding strikingly parallels the process of new meristem formation induced by antheridiogen removal. Moreover, ABA plays dual roles in gametophytes: It promotes cell fate re‐specification necessary for new meristem formation, while simultaneously reducing cell division activity within the meristem lineage. These two processes are independent of each other, but both rely on a functional ABA signaling pathway.

## RESULTS

### ABA overrides antheridiogen to trigger cell fate conversion and promote male‐to‐hermaphrodite conversion

**Figure 1:**
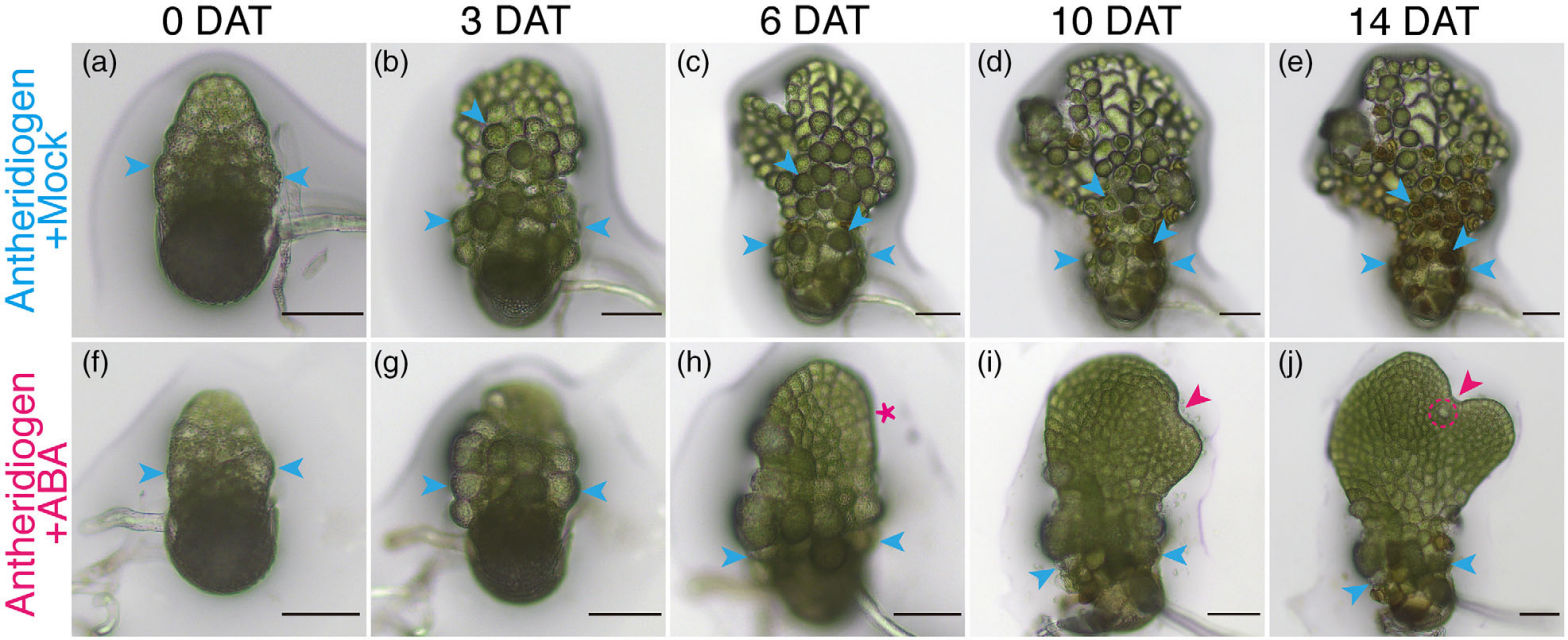
Live imaging of male gametophytes in the presence of antheridiogen alone or with ABA. (a–e) A representative male gametophyte grown on CFM with mock treatment remained male identity, with the majority of antheridia fully differentiated, matured, and ruptured. (f–j) A representative male gametophyte grown on CFM supplemented with 2.5 μm ABA converted into a hermaphrodite, characterized by forming the *de novo* meristem and archegonia. (a–j) At 2 DAG (a, f), male gametophytes were transferred onto fresh CFM containing either mock or ABA, and time‐lapse light micrographs were taken at the indicated days after treatment (DAT). (h) The magenta asterisk indicates the newly initiated proliferation site during the male‐to‐hermaphrodite conversion. (i, j) Magenta arrowheads indicate the *de novo* formation of a meristem, and the magenta dashed circle highlights the archegonium on the newly formed hermaphrodite. (a–j) Blue arrowheads indicate representative antheridia. Scale bars: 100 μm.

To assess the dynamic effects of ABA on Ceratopteris male development and sex‐type conversion, we performed long‐term live imaging using light microscopy to document detailed morphological changes following treatment (Figure 1a–j). For comparison, we also imaged normal hermaphrodite development and the previously characterized male‐to‐hermaphrodite conversion triggered by antheridiogen removal (Yang et al., 2025) (Figure S1a–h). Wild‐type (WT, Hn‐n) spores were surface‐sterilized and germinated on conditioned fern medium (CFM, containing antheridiogen) to ensure uniform male differentiation, or on fern medium (FM, without antheridiogen) to promote hermaphrodite development. At 2 days after germination (DAG), males with similar size were transferred to fresh CFM containing either mock treatment or 2.5 μm ABA, marking 0 days after treatment (0 DAT) (Figure 1a,f). In parallel, 2‐DAG hermaphrodites (Figure S1a) and 2‐DAG males (Figure S1e) were transferred to FM with mock treatment as additional controls. As expected, hermaphrodites on FM initiated a meristem notch and adjacent archegonia by 3 DAT (Figure S1b), with continued meristem proliferation, multiple archegonia formation, and substantial prothallus expansion by 10 DAT (Figure S1c,d). In the absence of antheridiogen, males on FM initiated a *de novo* meristem (Figure S1g) and subsequently formed archegonia (Figure S1h), completing male‐to‐hermaphrodite conversion by 10 DAT (Figure S1h), consistent with our earlier report (Yang et al., 2025). In contrast, in the presence of antheridiogen alone, male gametophytes maintained typical male characteristics and continually produced antheridia (Figure 1a–e). Over time, the majority of cells within the male body differentiated into sperm‐producing antheridia, and multiple antheridia reached maturity, ruptured, and released sperm (Figure 1c–e). By direct comparison, when exposed to both antheridiogen and ABA, the male gametophyte exhibited a gradual morphological transition (Figure 1f–j). At 3 DAT, the male was smaller and developed fewer antheridia (Figure 1g), compared to that with the mock (Figure 1b). By 6 DAT, a proliferative site emerged at the apical region (magenta asterisk, Figure 1h), which developed into a new meristem by 10 DAT (magenta arrowhead, Figure 1i). Continued proliferation led to the initiation of an archegonium (magenta dashed circle, Figure 1j) adjacent to the established meristem (magenta arrowhead, Figure 1j) at 14 DAT, ultimately resulting in a new hermaphrodite structure connected to the original male body (Figure 1j). Together, these findings show that ABA not only promotes the development of sex‐undetermined gametophytes into hermaphrodites as described previously (Hickok, 1983), but also enables fully committed males to undergo long‐term (over 14 days) conversion into hermaphrodites (Figure 1f–j). Thus, ABA overrides antheridiogen signaling, reprograms sexual fate, and robustly triggers *de novo* meristem formation in Ceratopteris gametophytes.

### Long‐term confocal imaging reveals cell division dynamics during ABA‐induced meristem formation

**Figure 2:**
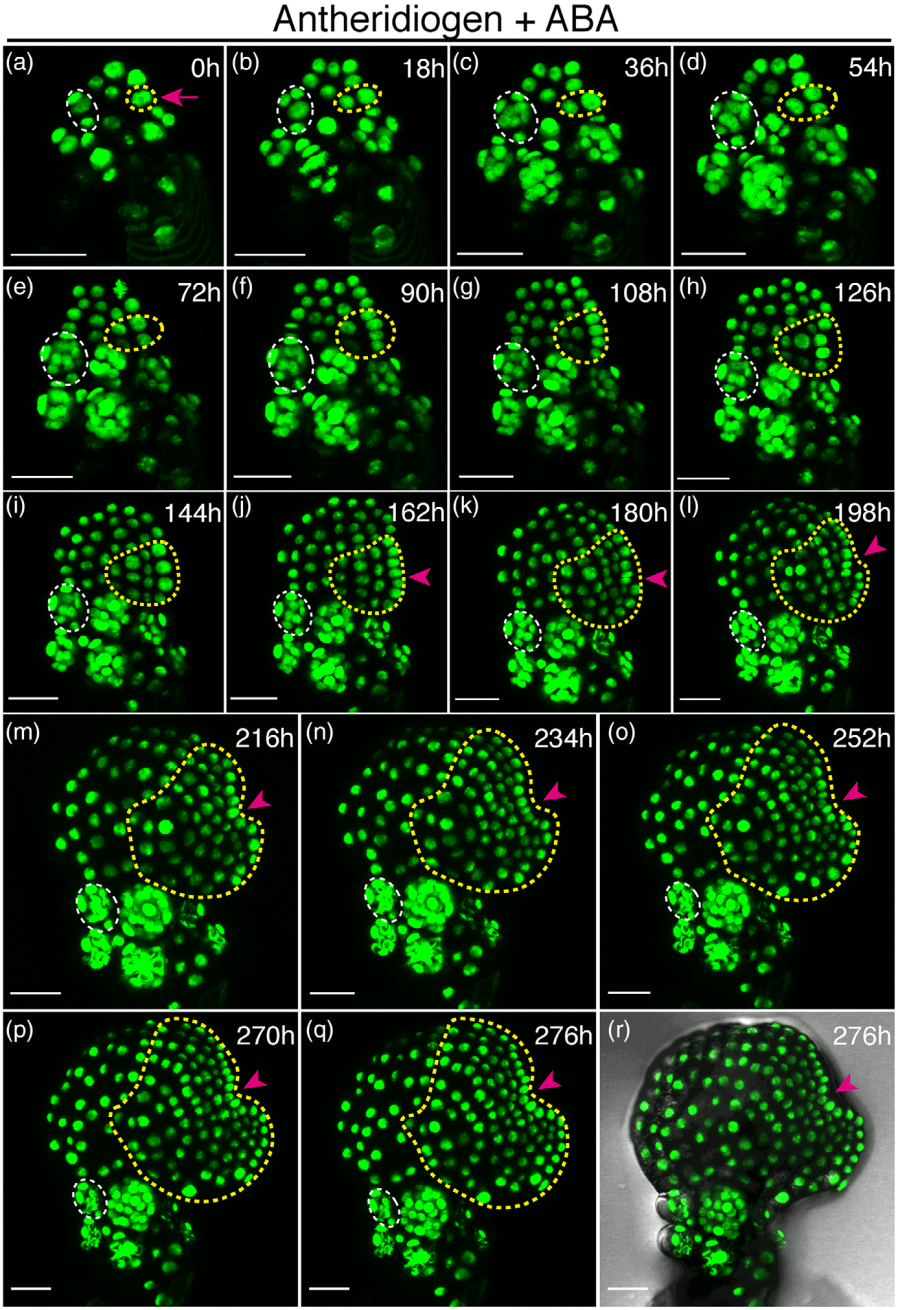
Time‐lapse confocal imaging of a male gametophyte in the presence of both antheridiogen and ABA. (a–r) Z‐projection views of a male gametophyte (Sample 3) expressing the *pCrUBQ10::H2B‐GFP::3'CrUBQ10* reporter. (a) At 2 DAG, the male gametophyte was transferred from CFM to CFM with 2.5 μm ABA and imaged at 0 h by laser scanning confocal microscopy. (a–r) Live imaging was performed every 6 h from 0 to 276 h. A magenta arrow in (a) marks the site of initial cell proliferation associated with *de novo* meristem formation. Yellow dashed outlines indicate the lineage of the meristem progenitor cell (MPC) across time points, and magenta arrowheads indicate the meristem notch. White dashed outlines highlight one representative antheridium. (a–q) show the GFP channel (green), and (r) shows a merged view of GFP and DIC channels at 276 h. Scale bars: 50 μm. At least three biological replicates grown in the presence of antheridiogen and ABA were live‐imaged under identical conditions at 6‐h intervals, all showing comparable results. The complete confocal image series for this sample from 0 to 276 h is provided in Figures S4–S6, and representative time points are shown here (Figure 2a–f correspond to Figure S4a,d,g,j,m,p; Figure 2g–k correspond to Figure S5c,f,i,l,o; Figure 2l–r correspond to Figure S6b,e,h,k,n,o,p). Full time series for the other two samples are provided in Figures S7–S9 and S10–S12, respectively.

**Figure 3:**
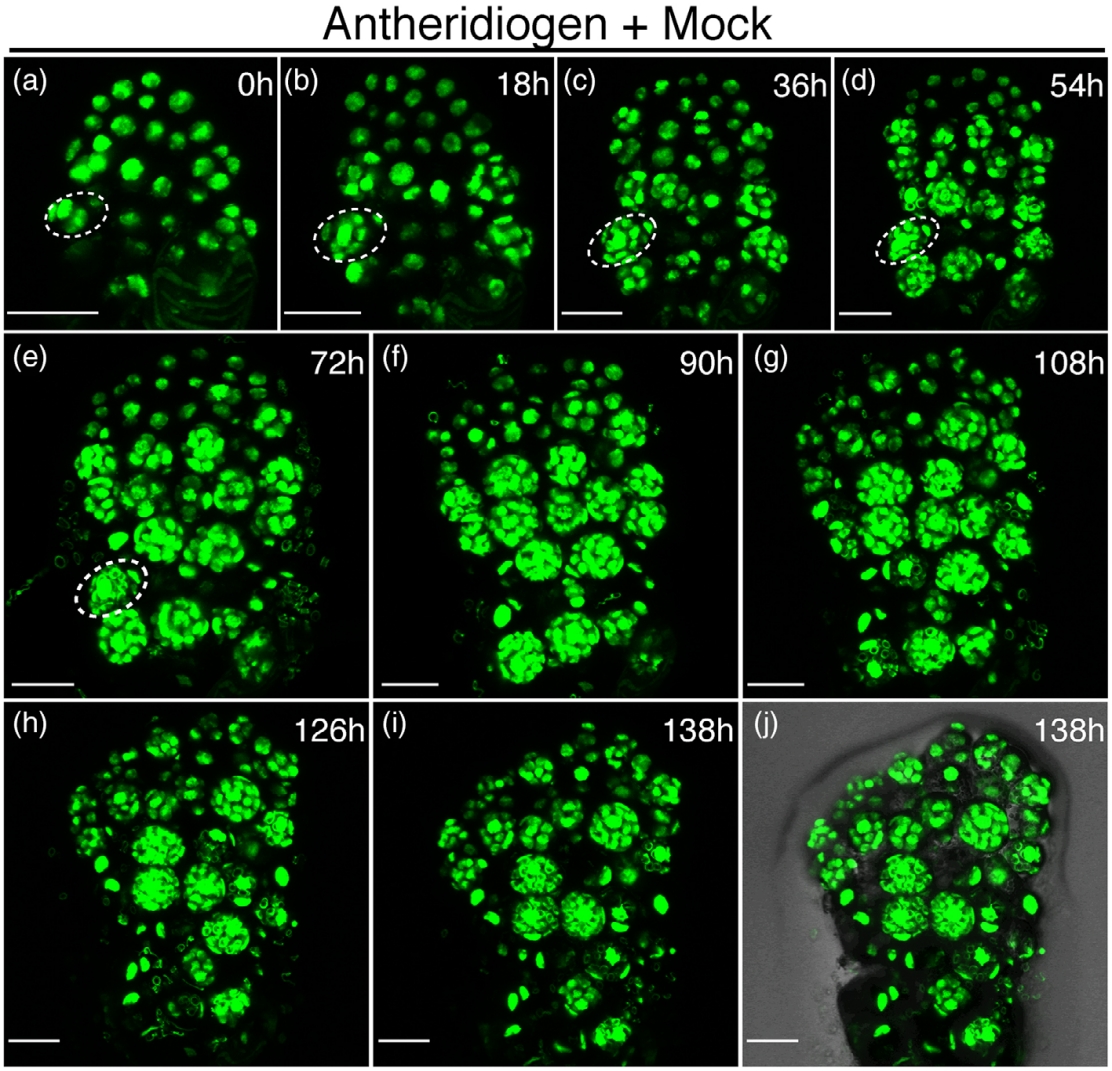
Time‐lapse confocal imaging of a male gametophyte in the presence of antheridiogen and mock treatment. (a–j) Z‐projection views of a male gametophyte (Mock Sample 5) expressing the *pCrUBQ10::H2B‐GFP::3'CrUBQ10* reporter. (a) At 2 DAG, the male gametophyte was transferred from CFM to CFM with mock treatment and imaged at 0 h by laser scanning confocal microscopy. (a–j) Live imaging was performed every 6 h from 0 to 138 h. White dashed circles highlight one representative antheridium. (a–i) show the GFP channel (green), and (j) shows a merged view of GFP and DIC channels at 138 h. Scale bars: 50 μm. At least three biological replicates grown on antheridiogen and mock were live‐imaged under identical conditions at 6‐h intervals, all showing comparable results. The complete confocal image series for this sample from 0 to 138 h is provided in Figures S13 and S14, and representative time points are shown here (Figure 3a–f correspond to Figure S13a,d,g,j,m,p; Figure 3g–j correspond to Figure S14c,f,h,i). Full time series for the other two samples are provided in Figures S15–S18, respectively.

To uncover the cellular basis of *de novo* meristem formation triggered by ABA, we then performed long‐term confocal time‐lapse imaging using the ubiquitously expressed nuclear marker H2B‐GFP (*pCrUBQ10::H2B‐GFP::3'CrUBQ10*) (Figures 2 and 3; Figures S2–S18). Transgenic spores were initially germinated on growth medium containing antheridiogen to induce male development. Subsequently, male gametophytes (2 DAG) were transferred to fresh medium containing both antheridiogen and 2.5 μm ABA (Figure 2; Figures S4–S6) or to medium containing antheridiogen with mock treatment (control; Figure 3; Figures S13 and S14). Non‐invasive confocal live imaging was carried out following the established procedure (Geng et al., 2022; Yang et al., 2025), which has been used in multiple sets of experiments and validated as not affecting overall morphology or developmental progression in Ceratopteris gametophytes (Geng et al., 2022; Yang et al., 2025). Gametophytes were imaged directly on growth medium at the initial time point (0 h) (Figure 2a; Figure S4a; Figure 3a, and Figure S13a) without mounting or dissection. Following each imaging session, samples were returned to identical growth conditions to maintain developmental consistency, and imaging was repeated every 6 h throughout the full time course (see methods for details).

Time lapse images revealed that, in response to ABA, male gametophytes underwent a slow but steady morphological transformation (Figure 2a–r; Figures S4a–p, S5a–p, and S6a–p). In contrast, mock‐treated controls continued normal male differentiation (Figure 3a–j; Figures S13a–p, and S14a–i), consistent with the light microscopy observations (Figure 1). Specifically, ABA delayed antheridium maturation and sperm release, as indicated by prolonged development within individual antheridia (white dashed circles, Figure 2; Figure S4–S6), whereas the mock‐treated control rapidly progressed male differentiation, completing antheridium initiation, maturation, and sperm release (Figure 3; Figure S13a–o). More importantly, ABA robustly triggered *de novo* meristem formation in male gametophytes (Figure 2; Figures S4–S6). An active proliferation site gradually emerged, marked by increasing nucleus number and division (Figure 2; Figures S4a–p and S5a–j), and a morphologically distinguishable meristem became evident by 156 h (magenta arrowhead, Figure S5K). This newly formed meristem continued to expand through sustained division and eventually formed a characteristic notch by 276 h (magenta arrowheads, Figure 2j–r, Figures S5k–p and S6a–p). All three independent male samples treated with both ABA and antheridiogen and examined in the time‐lapse imaging experiments exhibited consistent formation of multicellular meristems with notch structures (Figure 2; Figures S4–S12), indicating a conserved developmental trajectory. In contrast, no *de novo* meristem formation was observed in any mock‐treated male samples (Figure 3; Figures S13–S18), highlighting the distinct and specific developmental reprogramming induced by ABA.

### ABA induces de novo multicellular meristem formation from a single non‐antheridium cell

To determine the cellular origin of the ABA‐induced multicellular meristem, we performed lineage tracing based on sequential division events captured in time‐lapse confocal imaging. This analysis allowed us to reconstruct lineage relationships and trace the clonal expansion of progenitor cells over time. Strikingly, in all three independent ABA‐treated samples, the newly formed meristem (highlighted with yellow dashed lines; Figure 2; Figures S4–S12) consistently originated from a single non‐antheridium cell, designated as the meristem progenitor cell (MPC; Figure 2a; Figures S4a, S7a, and S10a, magenta arrows). The MPC progressively reentered the cell cycle and gave rise to an actively dividing lineage (yellow dashed lines; Figure 2a–i; Figures S4a–p, S5a–j, S7a–p, S8a–g, S10a–p, and S11a–h), which expanded dominantly over time. This lineage ultimately formed a new multicellular meristem composed entirely of MPC‐derived progeny (magenta arrowheads and yellow dashed lines; Figure 2j–r; Figures S5k–p, S6a–p, S8h‐p, S9a–o, S11i–p, and S12a–k). Interestingly, this single‐cell origin of the ABA‐induced meristem (Figure 2; Figures S4–S12) mirrors our previous finding that *de novo* meristems formed after antheridiogen removal also arise from a single MPC (Yang et al., 2025). These results suggest that two distinct signaling cues, ABA treatment and antheridiogen removal, converge on a conserved cellular mechanism that initiates new stem cell niche formation from a single progenitor cell.

### ABA triggers cell fate reprogramming but suppresses cell division during de novo meristem formation

**Figure 4:**
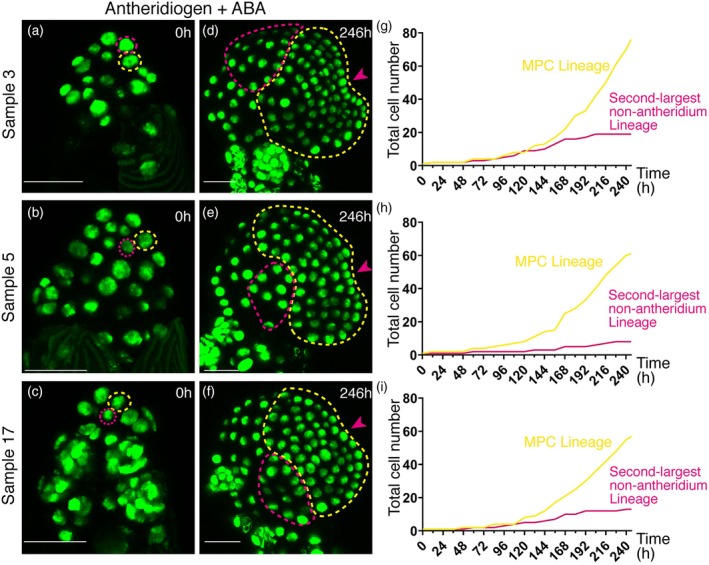
Quantitative analysis of cell number and division in the MPC lineages during ABA‐induced male‐to‐hermaphrodite conversion. (a–f) MPC lineages (highlighted with yellow dashed outlines) and the second‐largest non‐antheridium lineages (highlighted with magenta dashed outlines) in three independent samples at 0 h (a–c) and 246 h (d–f) of live imaging. Panels correspond to zoomed‐in views from other figures: (a), Figures 2a and S4a; (b), Figure S7a; (c), Figure S10a; (d), Figure S6j; (e), Figure S9j; (f), Figure S12j. Magenta arrowheads in (d–f) indicate the meristem notch. Scale bars: 50 μm. (g–i) Quantification and graphic presentation of total cell number in the MPC lineages (yellow) and the second‐largest non‐antheridium lineages (magenta) from the three samples at the indicated time points throughout the live‐imaging period. The source data files for panels (g–i) are included in Tables [Link], [Link], respectively.

We quantified total cell numbers in the MPC lineage, which represents the largest non‐antheridium lineage (yellow dashed outlines, Figure 4a–f; Figures S4–S12), at 12‐h intervals in all three ABA‐treated samples (Figure 4g–i; Tables [Link], [Link]). For direct comparison, we also identified the progression of other non‐antheridium lineages and quantified total cell numbers of the second‐largest non‐antheridium lineage (the largest non‐MPC lineage, magenta dashed outlines, Figure 4a–f) in the same samples (Figure 4g–i; Tables [Link], [Link]). This analysis revealed that cell division activity was strongly concentrated in the MPC lineage during ABA‐induced *de novo* meristem formation, whereas division outside the MPC lineage was greatly reduced. We then examined non‐antheridium lineages in mock‐treated samples (Figure S19). As expected, in the presence of antheridiogen but absence of ABA, most cells eventually differentiated into antheridia, and only a few non‐antheridium cell lineages could be identified by 60 h (Figure S19a–f). Quantification of these non‐antheridium lineages of the three mock‐treated male gametophytes showed only minimal rounds of cell division (Tables [Link], [Link]), in contrast to the robust divisions observed in the MPC lineages of ABA‐treated samples (Tables [Link], [Link]). These results confirm that ABA treatment, rather than mock treatment, induces cell fate re‐specification and establishment of the MPC lineage.

The cell number graphs further revealed that division within the ABA‐induced MPC lineage (yellow) proceeded slowly, characterized by an initial nearly quiescent phase followed by a gradual increase in proliferation activity (Figure 4g–i). Because all live imaging experiments were performed at 6‐h intervals using identical procedures, we compared these division dynamics (Figure 4g–i) with the MPC lineages previously characterized during *de novo* meristem formation after antheridiogen removal (Yang et al., 2025). This comparison (Table S7) showed striking differences between the two reprogramming conditions: in the presence of ABA and antheridiogen, cell division in the MPC lineage was markedly less active (an average of approximately 1.6 division events per 6‐h interval, Table S7), compared to more robust proliferation following antheridiogen removal (an average of approximately 5.2 division events per 6‐h interval, Table S7) (Yang et al., 2025). These findings align with earlier work (Hickok, 1983) and our light microscopy data (Figure 1b,g–j; Figure S1), which showed that ABA treatment resulted in hermaphrodites with considerably smaller sizes. Together, these results demonstrated that, in the presence of antheridiogen, ABA overrides antheridiogen signaling to reprogram cell fate and trigger *de novo* meristem formation, but concurrently limits cell division activity. As a consequence, the formation of a distinguishable meristem structure in ABA‐treated samples required substantially more time (Figures 2 and 3; Figures S4–S12, Table S7).

### ABA‐induced inhibition of cell division and cell fate reprogramming can be uncoupled, but both require functional ABA signaling during sex‐type conversion

**Figure 5:**
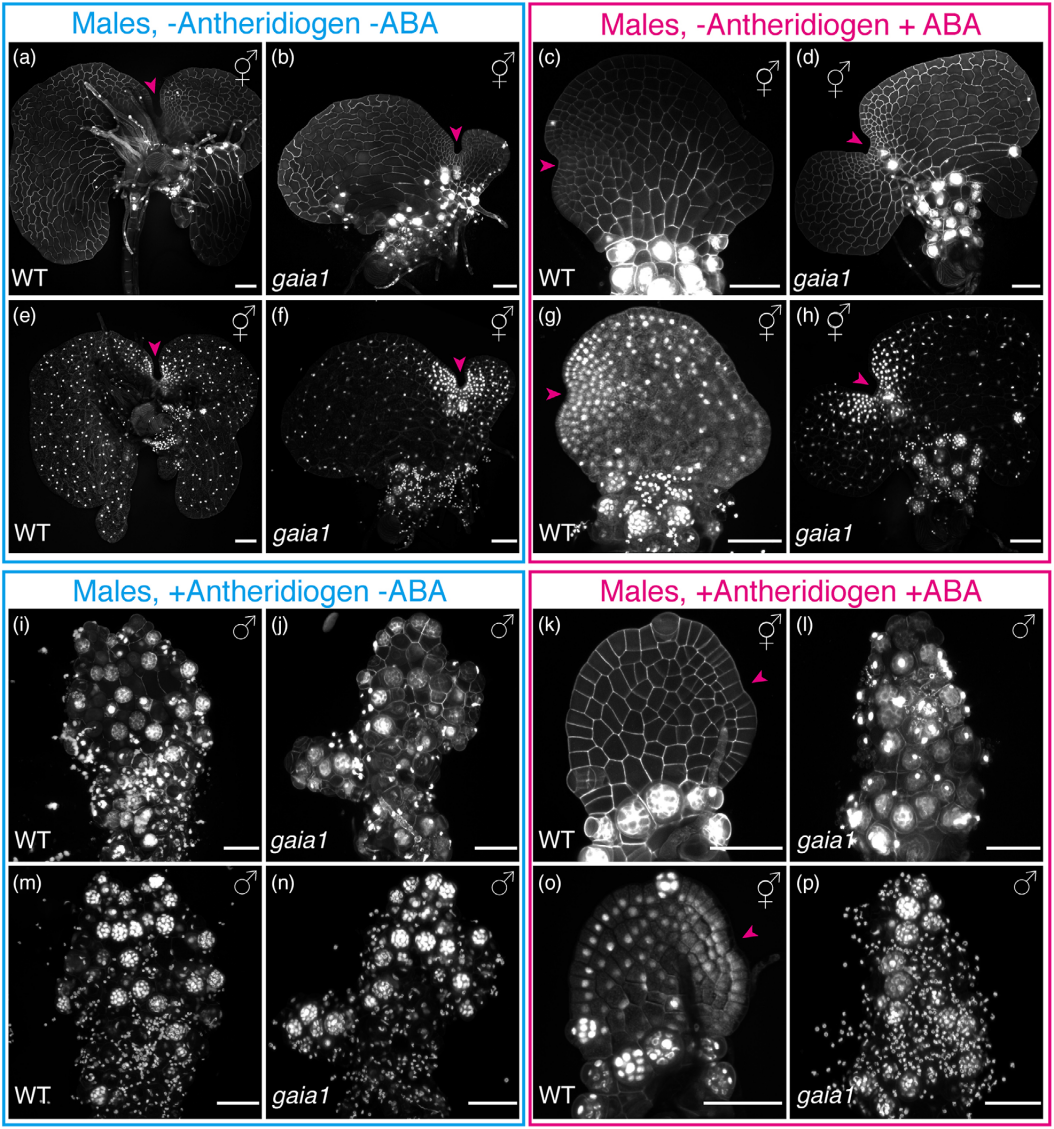
Confocal imaging of gametophytes of different genotypes under combinations of antheridiogen and ABA. (a–p) Confocal Z‐projection views of WT and *gaia1* gametophytes at 12 DAG (10 days after treatment). At 2 DAG, WT and *gaia1* males were transferred to four growth conditions: (1) without antheridiogen and without ABA (a, b, e, f), (2) without antheridiogen but with ABA (c, d, g, h), (3) with antheridiogen but without ABA (i, j, m, n), and (4) with both antheridiogen and ABA (k, l, o, p). (a–d, i–l) show cell outlines staining (gray), and (e–h, m–p) show subsequent nuclear staining of the same samples shown in (a–d, i–l), respectively (see Methods for details). Magenta arrowheads indicate newly formed meristem notches. Scale bars: 100 μm. Three biological replicates were examined for each genotype and treatment, with comparable results observed.

To further dissect the cellular and genetic basis of ABA‐induced cell fate reprogramming and cell division inhibition in Ceratopteris gametophytes, we examined *de novo* meristem formation in males under different treatment and genotype combinations. Specifically, we analyzed wild‐type (WT) and previously published *gaia1/ost1* mutant gametophytes (Mcadam et al., 2016) under four different growth conditions. Spores of both genotypes were surface‐sterilized and spread on CFM to induce male differentiation. At 2 DAG, germinated male gametophytes were transferred to one of four conditions: FM with mock treatment (no antheridiogen, no ABA), FM with 2.5 μm ABA (no antheridiogen, with ABA), CFM with mock treatment (antheridiogen, no ABA), or CFM with 2.5 μm ABA (antheridiogen and ABA). After 10 days of treatment (10 DAT, 12 DAG), gametophytes were first stained for cell outlines and imaged by confocal microscopy for high‐resolution morphological observation (Figure 5a–d,i–l), followed by nuclear staining and confocal imaging of the same samples (Figure 5e–h,m–p, see methods). As reported previously (Yang et al., 2025), removal of antheridiogen in WT triggered male‐to‐hermaphrodite conversion and the establishment of a new multicellular meristem (Figure 5a,e). By 10 DAT, the converted hermaphrodite showed substantial prothallus expansion and developed a deep concave notch (magenta arrowheads, Figure 5a,e). Interestingly, in the absence of antheridiogen but presence of ABA, *de novo* meristem formation still occurred in WT (Figure 5c,g); however, the resulting meristem was markedly smaller, with fewer cells and a shallower notch (magenta arrowheads, Figure 5c,g) compared with WT samples grown without antheridiogen and ABA (Figure 5a,e). In contrast, *gaia1* mutants exhibited robust male‐to‐hermaphrodite conversion under both no‐antheridiogen/no‐ABA and no‐antheridiogen/with‐ABA conditions, forming meristems with comparable size and morphology (Figure 5b,f,d,h). Additionally, in the absence of antheridiogen but presence of ABA, the *de novo* meristem formed in the *gaia1* background (Figure 5d,h) is substantially larger than that in WT (Figure 5c,g). These results indicate that during meristem initiation and development, ABA‐mediated suppression of cell proliferation is mechanistically independent of cell fate reprogramming and can occur without antheridiogen. Furthermore, under conditions with antheridiogen alone, both WT and *gaia1* gametophytes retained male identity and lacked meristems (Figure 5i,j,m,n). However, when both antheridiogen and ABA were present, WT males transitioned to hermaphrodites with a small meristem and fewer cells (Figure 5k,o), whereas *gaia1* gametophytes remained male and did not form a meristem (Figure 5l,p), consistent with prior findings (Mcadam et al., 2016). Together, these results demonstrate that ABA‐induced cell fate re‐specification and inhibition of cell division can occur independently in distinct developmental contexts, but both require a functional SnRK2‐mediated ABA signaling pathway.

## DISCUSSION

### ABA and antheridiogen in fern gametophytes

**Figure 6:**
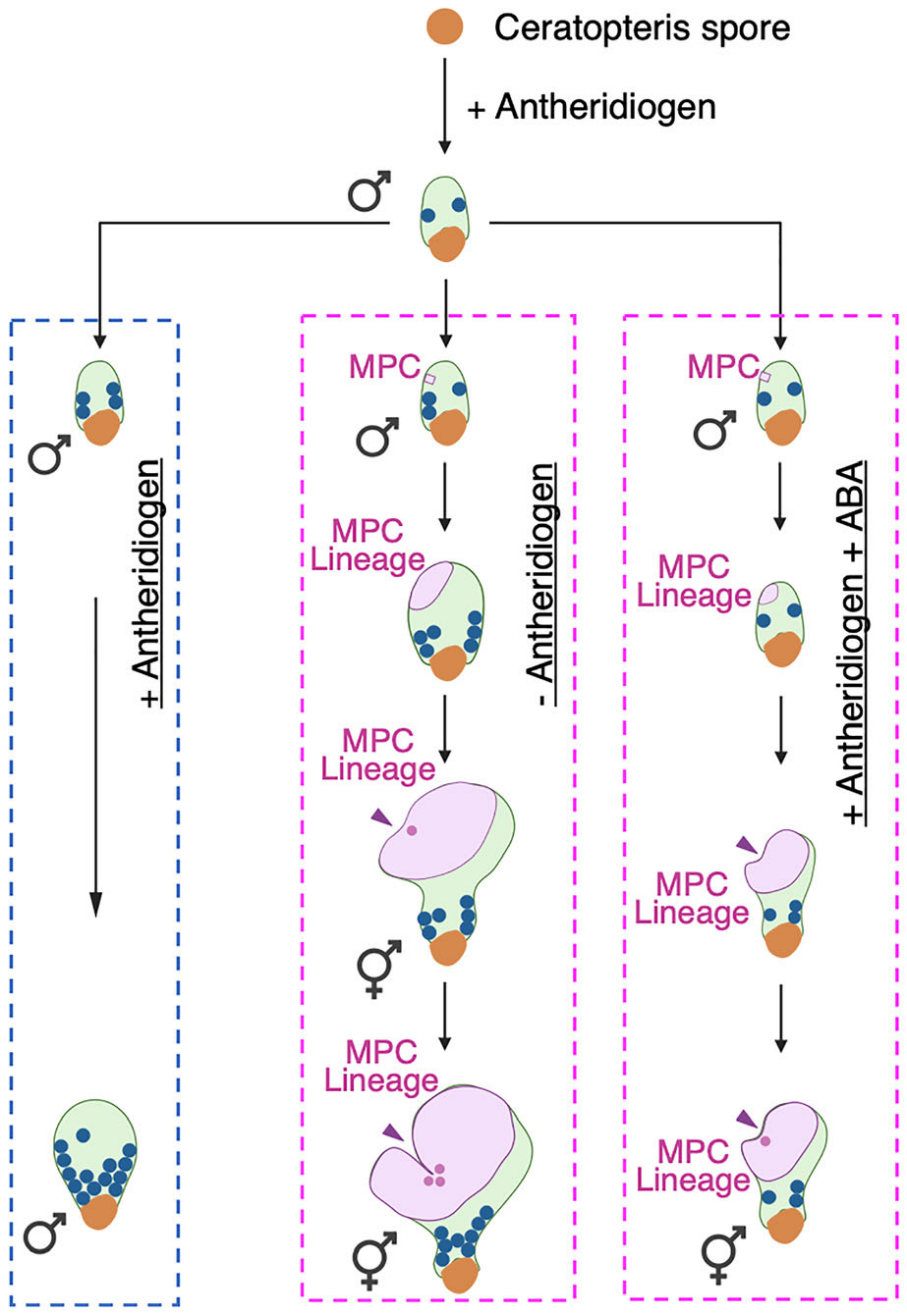
Diagrams illustrating Ceratopteris male gametophyte development and male‐to‐hermaphrodite conversion. In the presence of antheridiogen (cyan dashed box, left), germinated Ceratopteris spores develop into male gametophytes, which lack a meristem and initiate a few antheridia (blue dots). Continued exposure to antheridiogen maintains the male developmental program, leading to the formation of additional antheridia (blue dots), which eventually mature, rupture, and release sperm. In contrast, when antheridiogen is removed or depleted (magenta dashed box, middle), male gametophytes initiate a meristem progenitor cell (MPC, magenta). This cell undergoes active proliferation, and its progeny contribute to the formation of a *de novo* multicellular meristem (magenta arrowhead) and adjacent archegonia (magenta dots), marking the transition from male to hermaphrodite. In the presence of both antheridiogen and ABA (magenta dashed box, right), male gametophytes also initiate an MPC (magenta). However, cell division within the MPC lineage is markedly slower, producing a new multicellular meristem (magenta arrowhead) with delayed development. Under this condition, male‐to‐hermaphrodite conversion takes substantially longer, with gradual meristem development and eventual formation of adjacent archegonia (magenta dots) only after the meristem notch is established, later than in the conversion triggered by antheridiogen removal. The diagram was initially generated using BioRender and modified in PowerPoint.

This study uncovers the cellular dynamics underlying *de novo* meristem formation in response to ABA and antheridiogen. Our findings suggest that ABA plays two distinct roles in this developmental process. First, ABA overrides the effect of antheridiogen, which normally promotes male differentiation, and instead induces male‐to‐hermaphrodite conversion. This reprogramming allows specific cells to reenter the cell cycle and resume division, eventually giving rise to a new multicellular meristem with adjacent archegonia, both representing female traits. Second, ABA inhibits meristem cell division, reducing proliferation both within the MPC lineage and in newly established meristems. Interestingly, these two roles can be uncoupled: In ABA‐treated hermaphrodites, meristem proliferation is reduced, leading to smaller meristem size with fewer cells; in contrast, ABA‐treated males initiate the MPC lineage with active division and form new meristems, although proliferation is limited (Figure 6). Both effects are highly dependent on a conserved ABA signaling pathway.

Although this work focuses on ABA‐induced meristem formation, MPC lineage proliferation, and male‐to‐hermaphrodite conversion, our imaging also highlighted the antagonistic role of ABA and antheridiogen in antheridium formation and maturation (Figures 2 and 3), consistent with earlier reports (Hickok, 1983). Because antheridia form dynamic 3D structures at multiple developmental stages (Figures S4–S18), a dedicated study using advanced imaging and 3D analysis tools will be needed to fully resolve the cellular basis of antheridium development and sperm differentiation in response to ABA, antheridiogen, or both. Furthermore, an additional observation from this study is that, during male‐to‐hermaphrodite conversion, the prothallus size of hermaphrodites formed in the presence of both antheridiogen and ABA (Figure 5k,o) appears smaller than that of hermaphrodites exposed to ABA alone (Figure 5c,g). One interpretation is that antheridiogen sustains male identity, and the delayed antagonistic action of ABA on this program postpones sex‐type conversion, leading to prolonged growth. Alternatively, this may reflect combined effects of ABA and antheridiogen on cell proliferation during normal hermaphrodite development, which will be interesting to examine in future studies. Together, this work highlights ABA as both a reprogramming and inhibitory signal in *de novo* meristem formation and sex‐type conversion. Future studies dissecting the molecular signaling that mediates the antagonism between ABA and antheridiogen in gametophytes will provide more insight into fern sex determination, antheridium development, and meristem regulation.

### De novo meristem formation in Ceratopteris: Different triggers, conserved MPC lineages

Although distinct cues, removal of antheridiogen versus combined treatment of antheridiogen and ABA, create divergent developmental contexts, Ceratopteris male gametophytes consistently undergo *de novo* meristem formation in response to both (Figure 6). In each case, all meristematic cells can be traced back to a single non‐antheridium cell, termed the meristem progenitor cell (MPC). While the ABA‐induced MPC lineage divides more slowly than those triggered by antheridiogen removal, both exhibit comparable proliferation trajectories, including an initial quiescent phase followed by an active proliferation phase (Figure 4g–i, Table S7) (Yang et al., 2025). These findings suggest that different environmental signals converge on a shared cellular mechanism that reprograms cell fate, establishes the single MPC, and sustains lineage expansion to form a new meristem (Figure 6). This mechanism appears to differ from previously characterized *de novo* meristem formation in the sporophytes of seed plants. In model species such as Arabidopsis or tomato, several well‐studied processes, including shoot meristem regeneration from callus, initiation of axillary meristems, or lateral root formation, typically involve more than one founder or progenitor cells. These differences suggest a potentially lineage‐specific mechanism regulating multicellular meristem formation in fern gametophytes. Interestingly, in Ceratopteris hermaphrodites, targeted ablation of an established multicellular meristem can also induce the formation of a new meristem at a spatially distinct site outside the original meristem (Geng et al., 2022). It will be interesting to explore whether this regeneration process shares pathways similar to those involved in the *de novo* meristem formation observed during male‐to‐hermaphrodite conversion. Furthermore, a recent study revealed repeated *de novo* meristem formation in an epiphytic fern species, enabling the production of multiple prothalli from a single gametophyte and contributing to clonal growth and extended longevity (Wu et al., 2025). Whether single‐cell‐derived multicellular meristems represent a unique feature of Ceratopteris or reflect a broader conserved strategy among ferns remains a noteworthy question for future research.

## MATERIAL AND METHODS

### Plant materials and growth conditions

Spores of *Ceratopteris richardii* Hn‐n wild‐type (Hickok et al., 1987), *pCrUBQ10::H2B‐GFP::3'CrUBQ10* transgenic lines (Yang et al., 2025), and the *gaia1/ost1* mutant (Mcadam et al., 2016) were surface‐sterilized and spread onto Petri dishes of conditioned fern medium (CFM), which contained antheridiogen and was prepared as described previously (Banks et al., 1993). Specifically, *her‐19* spores (Banks, 1994; Banks et al., 1993) were surface‐sterilized and cultured in liquid FM (0.1 g/1 L) with shaking at 110 rpm for 1 month in a growth chamber. Then, the liquid medium was filtered and supplemented with 0.5× MS salts with vitamins (PhytoTechnology Laboratories, Lenexa, KS, USA), adjusted to pH 6.0, and solidified with 0.7% (w/v) agar (Sigma‐Aldrich, Saint Louis, MI, USA) to prepare CFM. Spores and germinated gametophytes were cultured under continuous light at 29°C in growth chambers (Percival, Perry, lowa, USA).

### Light microscopy

Male gametophyte development in the presence of antheridiogen alone or in combination with ABA was assessed through live imaging (Figure 1). Male‐to‐hermaphrodite conversion induced by antheridiogen removal and hermaphrodite development were also imaged as controls (Figure S1). Ceratopteris Hn‐n spores were surface‐sterilized and inoculated on CFM to induce the male developmental program or on FM to promote hermaphrodite development. At 2 DAG, male gametophytes of similar size were randomly selected and transferred to fresh CFM plates containing either mock treatment (0.1% ethanol, 0 μm ABA) or 2.5 μm ABA (in 0.1% ethanol), or to fresh FM plates (no antheridiogen) with mock treatment. At 2 DAG, hermaphrodites of similar size were randomly selected and transferred to fresh FM plates containing mock treatment for live imaging. All samples were cultured under identical conditions in a Percival growth chamber. At least four independent gametophyte samples were imaged for each treatment at the indicated days after treatment (DAT) using an Olympus CKX53 microscope equipped with a Mlchrome 5 Pro digital camera. Representative samples for each treatment are shown in Figure 1 and Figure S1.

### Confocal microscopy

Non‐invasive confocal time‐lapse imaging was performed following a previously established procedure (Geng et al., 2022), with minor modifications. To assess cellular dynamics in male gametophytes exposed to either antheridiogen alone or in combination with ABA, spores of Ceratopteris expressing the *pCrUBQ10::H2B‐GFP::3'CrUBQ10* reporter (Yang et al., 2025) were surface‐sterilized and spread on CFM (containing antheridiogen) to initiate the male program. At 2 DAG, male gametophytes were transferred to either CFM supplemented with 2.5 μm ABA (Figure 2; Figures S4–S12) or CFM with mock treatment (Figure 3; Figures S13–S18) and imaged directly on the plates using confocal microscopy as the initial time point (0 h), without mounting or dissection. After each imaging session, the plates were returned to a growth chamber located adjacent to the confocal microscope to maintain consistent growth conditions. Live imaging of samples grown with both antheridiogen and ABA was performed at 6‐h intervals until a well‐defined *de novo* meristem formed (Figure 2; Figures S4–S12). The entire confocal time‐lapse series captured the dynamic process of *de novo* meristem initiation and establishment but did not extend to the later stage of archegonium formation (Figure 2; Figures S4–S12). As a control, live imaging of male gametophytes grown with antheridiogen and mock treatment was also performed at 6‐h intervals, continuing until most antheridia fully developed and ruptured (Figure 3; Figures S13–S18).

Spores of Ceratopteris WT and *gaia1* were surface‐sterilized and plated on CFM. At 2 DAG, male gametophytes were transferred to either FM (no antheridiogen) or CFM (with antheridiogen) containing mock treatment (0.1% ethanol, no ABA) or 2.5 μm ABA (in 0.1% ethanol). At 10 days after treatment, samples were stained with Propidium iodide (PI), rinsed with sterilized water, and imaged by confocal microscopy to visualize cell outlines. Subsequently, the same samples were treated with 100% ethanol for 1 min, stained again with PI for 1–2 min, rinsed with sterilized water, and imaged again to visualize nuclei (Figure 5).

All imaging was performed on an upright ZEISS LSM 880 laser scanning confocal microscope using ZEN Black software, following the procedures and settings described in previous studies (Geng et al., 2022; Geng & Zhou, 2019; Wu et al., 2021; Wu, Yan, Yang, et al., 2022; Yang et al., 2025). Gametophytes were directly imaged on Petri dishes with a Plan‐Apochromat 10×/0.45 objective lens using 1.0 μm z‐intervals, with frame sizes ranging from 512 × 512 to 760 × 760 pixels. GFP was excited with a 488 nm laser line, and emissions were collected from 491 to 562 nm. The transmission channel (DIC) was also excited with the 488 nm laser line, and signals were captured through T‐PMT to visualize gametophyte cell outlines. PI‐stained samples were excited with a 561 nm laser line, and emissions were collected from 569 to 620 nm. Confocal z‐stacks were processed as maximum‐intensity Z projections using Fiji/ImageJ. When needed, image brightness was uniformly adjusted using ImageJ across the entire frame to ensure clear nuclear visualization.

### Cell lineages and cell number quantifications

The MPC lineage (yellow dashed outlines) and the second‐largest non‐antheridium lineage (magenta dashed outlines) in samples grown in the presence of both ABA and antheridiogen (Figure 4) were identified based on cell divisions observed over time. Similarly, non‐antheridium cell lineages in male samples grown in the presence of antheridiogen and mock treatment were also identified based on cell division events over time. Total cell numbers of these lineages were quantified from confocal images at the indicated time points (Tables [Link], [Link]). In the presence of antheridiogen but absence of ABA, most cells eventually differentiated into antheridia. Because antheridia form complex 3D structures, they were not included in any of the cell number quantifications in this study. Quantification of cell numbers in the MPC lineages (Table S7) from the samples following antheridiogen removal was based on published images and analyses (Yang et al., 2025).

## AUTHOR CONTRIBUTIONS

XY and YZ conceived the research direction; YZ supervised the research progress; XY performed experiments; XY, XL, and YZ discussed and interpreted experimental results; XY performed quantitative analysis; XY and YZ wrote the manuscript, and XL revised the manuscript. All the authors read and approved the manuscript.

## CONFLICT OF INTEREST

The authors declare no competing interests.

## Supporting information


**Figure S1.** Live imaging of hermaphrodites and males in the absence of antheridiogen with mock treatment.
**Figure S2.** Confocal imaging of a Ceratopteris male gametophyte expressing the *pCrUBQ10::H2B‐GFP::3'CrUBQ10* nuclear marker.
**Figure S3.** Confocal imaging of a Ceratopteris hermaphrodite gametophyte expressing the *pCrUBQ10::H2B‐GFP::3'CrUBQ10* nuclear marker.
**Figure S4.** Time‐lapse confocal imaging of a male gametophyte from 0 to 90 h in the presence of both antheridiogen and ABA.
**Figure S5.** Time‐lapse confocal imaging of the first male gametophyte (Sample 3) from 96 to 186 h reveals *de novo* meristem development in the presence of both antheridiogen and ABA.
**Figure S6.** Continued time‐lapse imaging of the first male gametophyte (Sample 3) from 192 to 276 h shows meristem notch formation in the presence of both antheridiogen and ABA.
**Figure S7.** Time‐lapse confocal imaging of the second male gametophyte (Sample 5) from 0 to 90 h in the presence of both antheridiogen and ABA.
**Figure S8.** Time‐lapse confocal imaging of the second male gametophyte (Sample 5) from 96 to 186 h reveals de novo meristem development in the presence of both antheridiogen and ABA.
**Figure S9.** Continued time‐lapse imaging of the second male gametophyte (Sample 5) shows meristem notch formation in the presence of both antheridiogen and ABA.
**Figure S10.** Time‐lapse confocal imaging of the third male gametophyte (Sample 17) from 0 to 90 h in the presence of both antheridiogen and ABA.
**Figure S11.** Time‐lapse confocal imaging of the third male gametophyte (Sample 17) from 96 to 186 h reveals de novo meristem development in the presence of both antheridiogen and ABA.
**Figure S12.** Continued time‐lapse imaging of the third male gametophyte (Sample 17) shows meristem notch formation in the presence of both antheridiogen and ABA.
**Figure S13.** Time‐lapse confocal imaging of a male gametophyte from 0 to 90 h in the presence of antheridiogen alone.
**Figure S14.** Continued time‐lapse imaging of the first mock sample (Mock Sample 5) from 96 to 138 h reveals antheridium maturation in the presence of antheridiogen alone.
**Figure S15.** Time‐lapse confocal imaging of the second male gametophyte (Mock Sample 1) from 0 to 90 h in the presence of antheridiogen alone.
**Figure S16.** Continued time‐lapse imaging of the second sample (Mock Sample 1) from 96 to 138 h reveals antheridium maturation in the presence of antheridiogen alone.
**Figure S17.** Time‐lapse confocal imaging of the third male gametophyte (Mock Sample 2) from 0 to 90 h in the presence of antheridiogen alone.
**Figure S18.** Continued time‐lapse imaging of the third sample (Mock Sample 2) from 96 to 138 h reveals antheridium maturation in the presence of antheridiogen alone.
**Figure S19.** Non‐antheridium cell lineages from three male gametophytes in the presence of antheridiogen alone.

**Table S1.** Source Data for Figure 4g. Total cell counts of the MPC lineage (yellow) and the second‐largest non‐antheridium lineage (magenta) in Sample 3, at various time points in the presence of both antheridiogen and ABA.

**Table S2.** Source Data for Figure 4h. Total cell counts of the MPC lineage (yellow) and the second‐largest non‐antheridium lineage (magenta) in Sample 5, at various time points in the presence of both antheridiogen and ABA.

**Table S3.** Source Data for Figure 4i. Total cell counts of the MPC lineage (yellow) and the secondlargest non‐antheridium lineage (magenta) in Sample 17, at various time points in the presence of both antheridiogen and ABA.

**Table S4.** Total cell counts of the non‐antheridium lineages (yellow and magenta) in mock‐treated Sample 5, at various time points (0‐60 h) in the presence of antheridiogen alone.

**Table S5.** Total cell counts of the non‐antheridium lineages (yellow and magenta) in mock‐treated Sample 1, at various time points (0‐60 h) in the presence of antheridiogen alone.

**Table S6.** Total cell counts of the non‐antheridium lineage (yellow) in mock‐treated Sample 2, at various time points (0‐60 h) in the presence of antheridiogen alone.

**Table S7.** Quantitative comparison of cell division events in the MPC lineage (yellow) during male‐to‐hermaphrodite conversion, triggered either by antheridiogen removal or by ABA treatment.

## Data Availability

All data are included in the manuscript text, figures, or supporting information.

## References

[tpj70543-bib-0001] Atallah, N.M. & Banks, J.A. (2015) Reproduction and the pheromonal regulation of sex type in fern gametophytes. Frontiers in Plant Science, 6, 100. 25798139 10.3389/fpls.2015.00100 PMC4351586

[tpj70543-bib-0002] Banks, J.A. (1994) Sex‐determining genes in the homosporous fern Ceratopteris. Development (Cambridge, England), 120, 1949–1958. 7925000 10.1242/dev.120.7.1949

[tpj70543-bib-0003] Banks, J.A. (1999) Gametophyte development in ferns. Annual Review of Plant Biology, 50, 163–186. 10.1146/annurev.arplant.50.1.163 15012207

[tpj70543-bib-0004] Banks, J.A. , Hickok, L. & Webb, M.A. (1993) The programming of sexual phenotype in the homosporous fern *Ceratopteris richardii* . International Journal of Plant Sciences, 154, 522–534.

[tpj70543-bib-0005] Bowman, J.L. , Sakakibara, K. , Furumizu, C. & Dierschke, T. (2016) Evolution in the cycles of life. Annual Review of Genetics, 50, 133–154. 10.1146/annurev-genet-120215-035227 27617970

[tpj70543-bib-0006] Bui, L.T. , Cordle, A.R. , Irish, E.E. & Cheng, C.‐L. (2015) Transient and stable transformation of Ceratopteris richardii gametophytes. BMC Research Notes, 8, 1–10. 26040630 10.1186/s13104-015-1193-x PMC4467839

[tpj70543-bib-0007] Burow, K.M. , Yang, X. , Zhou, Y. , Dilkes, B.P. & Wisecaver, J.H. (2025) A BRASSINOSTEROID INSENSISTIVE 1 receptor kinase ortholog is required for sex determination in *Ceratopteris richardii* . The Plant Cell, 37, koaf058. 40341930 10.1093/plcell/koaf058 PMC12063094

[tpj70543-bib-0008] Cai, S. , Chen, G. , Wang, Y. , Huang, Y. , Marchant, D.B. , Wang, Y. et al. (2017) Evolutionary conservation of ABA signaling for stomatal closure. Plant Physiology, 174, 732–747. 28232585 10.1104/pp.16.01848 PMC5462018

[tpj70543-bib-0009] Cheruiyot, D.J. & Schwartz, B.W. (2007) Conversion of male gametophytes to hermaphrodites in the fern *Ceratopteris richardii* . Bios, 78, 58–61.

[tpj70543-bib-0010] Conway, S.J. & Di Stilio, V.S. (2020) An ontogenetic framework for functional studies in the model fern *Ceratopteris richardii* . Developmental Biology, 457, 20–29. 31470018 10.1016/j.ydbio.2019.08.017

[tpj70543-bib-0011] Cutler, S.R. , Rodriguez, P.L. , Finkelstein, R.R. & Abrams, S.R. (2010) Abscisic acid: emergence of a core signaling network. Annual Review of Plant Biology, 61, 651–679. 10.1146/annurev-arplant-042809-112122 20192755

[tpj70543-bib-0012] Eberle, J.R. & Banks, J.A. (1996) Genetic interactions among sex‐determining genes in the fern *Ceratopteris richardii* . Genetics, 142, 973–985. 8849903 10.1093/genetics/142.3.973 PMC1207034

[tpj70543-bib-0013] Finch‐Savage, W.E. & Leubner‐Metzger, G. (2006) Seed dormancy and the control of germination. New Phytologist, 171, 501–523. 16866955 10.1111/j.1469-8137.2006.01787.x

[tpj70543-bib-0014] Furber, M. & Mander, L.N. (1988) Synthesis and confirmation of structure of the antheridium‐inducing factor from the fern *Anemia mexicana* . Journal of the American Chemical Society, 110, 4084–4085.

[tpj70543-bib-0015] Gaillochet, C. & Lohmann, J.U. (2015) The never‐ending story: from pluripotency to plant developmental plasticity. Development, 142, 2237–2249. 26130755 10.1242/dev.117614 PMC4510588

[tpj70543-bib-0016] Geng, Y. , Cai, C. , Mcadam, S.A. , Banks, J.A. , Wisecaver, J.H. & Zhou, Y. (2021) A de novo transcriptome assembly of Ceratopteris richardii provides insights into the evolutionary dynamics of complex gene families in land plants. Genome Biology and Evolution, 13, evab042. 33681974 10.1093/gbe/evab042 PMC7975763

[tpj70543-bib-0017] Geng, Y. , Xie, C. , Yan, A. et al. (2024) A conserved GRAS‐domain transcriptional regulator links meristem indeterminacy to sex determination in Ceratopteris gametophytes. Current Biology, 34, 3454–3472.e7. 39059395 10.1016/j.cub.2024.06.064 PMC11364212

[tpj70543-bib-0018] Geng, Y. , Xie, C. , Zhang, C. , Liu, X. & Zhou, Y. (2025) Functions and regulation of HAM family genes in meristems during gametophyte and sporophyte generations. Plant, Cell & Environment, 48, 2125–2131. 10.1111/pce.15286 PMC11788942 39558470

[tpj70543-bib-0019] Geng, Y. , Yan, A. & Zhou, Y. (2022) Positional cues and cell division dynamics drive meristem development and archegonium formation in Ceratopteris gametophytes. Communications Biology, 5, 650. 35778477 10.1038/s42003-022-03627-y PMC9249879

[tpj70543-bib-0020] Geng, Y. & Zhou, Y. (2019) Confocal live imaging of shoot apical meristems from different plant species. JoVE, Journal of Visualized Experiments, 145, e59369. 10.3791/59369 30985746

[tpj70543-bib-0021] Granados, S. , Rivera, A. , Cañal, M.J. & Fernández, H. (2022) The Effect of Phytohormones and Inhibitors on Apogamous Gametophytes of *Dryopteris affinis* ssp. *affinis* . In: Ferns: Biotechnology, Propagation, Medicinal Uses and Environmental Regulation. Singapore: Springer Nature Singapore, pp. 325–342.

[tpj70543-bib-0022] Han, H. , Liu, X. & Zhou, Y. (2020) Transcriptional circuits in control of shoot stem cell homeostasis. Current Opinion in Plant Biology, 53, 50–56. 31766002 10.1016/j.pbi.2019.10.004

[tpj70543-bib-0023] Harrison, C.J. (2017) Development and genetics in the evolution of land plant body plans. Philosophical Transactions of the Royal Society, B: Biological Sciences, 372, 20150490. 10.1098/rstb.2015.0490 PMC5182422 27994131

[tpj70543-bib-0024] Heidstra, R. & Sabatini, S. (2014) Plant and animal stem cells: similar yet different. Nature Reviews Molecular Cell Biology, 15, 301–312. 24755933 10.1038/nrm3790

[tpj70543-bib-0025] Hickok, L.G. (1983) Abscisic acid blocks antheridiogen‐induced antheridium formation in gametophytes of the fern Ceratopteris. Canadian Journal of Botany, 61, 888–892.

[tpj70543-bib-0026] Hickok, L.G. , Warne, T.R. & Fribourg, R.S. (1995) The biology of the fern Ceratopteris and its use as a model system. International Journal of Plant Sciences, 156, 332–345.

[tpj70543-bib-0027] Hickok, L.G. , Warne, T.R. & Slocum, M.K. (1987) Ceratopteris richardii: applications for experimental plant biology. American Journal of Botany, 74, 1304–1316.

[tpj70543-bib-0028] Hsu, P.K. , Dubeaux, G. , Takahashi, Y. & Schroeder, J.I. (2021) Signaling mechanisms in abscisic acid‐mediated stomatal closure. The Plant Journal, 105, 307–321. 33145840 10.1111/tpj.15067 PMC7902384

[tpj70543-bib-0029] Imaichi, R. (2013) A new classification of the gametophyte development of homosporous ferns, focusing on meristem behaviour. Fern Gazette, 19, 141–156.

[tpj70543-bib-0030] Juarez, C. & Banks, J.A. (1998) Sex determination in plants. Current Opinion in Plant Biology, 1, 68–72. 10066559 10.1016/s1369-5266(98)80130-1

[tpj70543-bib-0031] Kaźmierczak, A. (2007) Ethylene is a modulator of gibberellic acid‐induced antheridiogenesis in *Anemia phyllitidis* gametophytes. Biologia Plantarum, 51, 683–689.

[tpj70543-bib-0032] Kean‐Galeno, T. , Lopez‐Arredondo, D. & Herrera‐Estrella, L. (2024) The shoot apical meristem: an evolutionary molding of higher plants. International Journal of Molecular Sciences, 25, 1519. 38338798 10.3390/ijms25031519 PMC10855264

[tpj70543-bib-0033] Kinosian, S.P. & Wolf, P.G. (2022) The biology of C. richardii as a tool to understand plant evolution. eLife, 11, e75019. 35311640 10.7554/eLife.75019 PMC8979586

[tpj70543-bib-0034] Kitagawa, M. & Jackson, D. (2019) Control of meristem size. Annual Review of Plant Biology, 70, 269–291. 10.1146/annurev-arplant-042817-040549 31035828

[tpj70543-bib-0035] Leung, J. & Giraudat, J. (1998) Abscisic acid signal transduction. Annual Review of Plant Biology, 49, 199–222. 10.1146/annurev.arplant.49.1.199 15012233

[tpj70543-bib-0036] Li, W. & Ma, H. (2002) Gametophyte development. Current Biology, 12, R718‐R21. 12419198 10.1016/s0960-9822(02)01245-9

[tpj70543-bib-0037] Liu, X. & Hou, X. (2018) Antagonistic regulation of ABA and GA in metabolism and signaling pathways. Frontiers in Plant Science, 9, 251. 29535756 10.3389/fpls.2018.00251 PMC5834473

[tpj70543-bib-0038] Marchant, D.B. , Chen, G. , Cai, S. , Chen, F. , Schafran, P. , Jenkins, J. et al. (2022) Dynamic genome evolution in a model fern. Nature Plants, 8, 1038–1051. 36050461 10.1038/s41477-022-01226-7 PMC9477723

[tpj70543-bib-0039] Mcadam, S.A. & Brodribb, T.J. (2012) Stomatal innovation and the rise of seed plants. Ecology Letters, 15, 1–8. 22017636 10.1111/j.1461-0248.2011.01700.x

[tpj70543-bib-0040] Mcadam, S.A. , Brodribb, T.J. , Banks, J.A. , McAdam, S.A.M. , Hedrich, R. , Atallah, N.M. et al. (2016) Abscisic acid controlled sex before transpiration in vascular plants. Proceedings of the National Academy of Sciences of the United States of America, 113, 12862–12867. 27791082 10.1073/pnas.1606614113 PMC5111647

[tpj70543-bib-0041] Mccormick, S. (2004) Control of male gametophyte development. The Plant Cell, 16, S142–S153. 15037731 10.1105/tpc.016659 PMC2643393

[tpj70543-bib-0042] Meyerowitz, E.M. (1997) Genetic control of cell division patterns in developing plants. Cell, 88, 299–308. 9039256 10.1016/s0092-8674(00)81868-1

[tpj70543-bib-0043] Näf, U. , Nakanishi, K. & Endo, M. (1975) On the physiology and chemistry of fern antheridiogens. The Botanical Review, 41, 315–359.

[tpj70543-bib-0044] Nayar, B. & Kaur, S. (1971) Gametophytes of homosporous ferns. The Botanical Review, 37, 295–396.

[tpj70543-bib-0045] Plackett, A.R. , Di Stilio, V.S. & Langdale, J.A. (2015) Ferns: the missing link in shoot evolution and development. Frontiers in Plant Science, 6, 972. 26594222 10.3389/fpls.2015.00972 PMC4635223

[tpj70543-bib-0046] Rensing, S.A. (2017) Why we need more non‐seed plant models. New Phytologist, 216, 355–360. 28191633 10.1111/nph.14464

[tpj70543-bib-0047] Rivera, A. , Cañal, M.J. , Grossniklaus, U. & Fernández, H. (2018) The gametophyte of fern: born to reproduce. In: Current advances in fern research. Cham: Springer International Publishing, pp. 3–19.

[tpj70543-bib-0048] Shpak, E.D. & Uzair, M. (2025) WUSCHEL: the essential regulator of the Arabidopsis shoot apical meristem. Current Opinion in Plant Biology, 85, 102739. 40381531 10.1016/j.pbi.2025.102739

[tpj70543-bib-0049] Shu, K. , Zhou, W. , Chen, F. , Luo, X. & Yang, W. (2018) Abscisic acid and gibberellins antagonistically mediate plant development and abiotic stress responses. Frontiers in Plant Science, 9, 416. 29636768 10.3389/fpls.2018.00416 PMC5881240

[tpj70543-bib-0050] Song, L. , Huang, S.‐S.C. , Wise, A. , Castanon, R. , Nery, J.R. , Chen, H. et al. (2016) A transcription factor hierarchy defines an environmental stress response network. Science, 354, aag1550. 27811239 10.1126/science.aag1550 PMC5217750

[tpj70543-bib-0051] Takeno, K. , Yamane, H. , Yamauchi, T. , Takahashi, N. , Furber, M. & Mander, L.N. (1989) Biological activities of the methyl ester of gibberellin a73, a novel and principal antheridiogen in *Lygodium japonicum* . Plant and Cell Physiology, 30, 201–205.

[tpj70543-bib-0052] Tanaka, J. , Yano, K. , Aya, K. , Hirano, K. , Takehara, S. , Koketsu, E. et al. (2014) Antheridiogen determines sex in ferns via a spatiotemporally split gibberellin synthesis pathway. Science, 346, 469–473. 25342803 10.1126/science.1259923

[tpj70543-bib-0053] Thomas, T. , Wareing, P. & Robinson, P.M. (1964) Action of the sycamore dormin as a gibberellin antagonist.

[tpj70543-bib-0054] Warne, T.R. & Hickok, L.G. (1989) Evidence for a gibberellin biosynthetic origin of Ceratopteris antheridiogen. Plant Physiology, 89, 535–538. 16666578 10.1104/pp.89.2.535 PMC1055877

[tpj70543-bib-0055] Warne, T.R. & Hickok, L.G. (1991) Control of sexual development in gametophytes of *Ceratopteris richardii*: antheridiogen and abscisic acid. Botanical Gazette, 152, 148–153.

[tpj70543-bib-0056] Woudenberg, S. , Alvarez, M.D. , Rienstra, J. , Levitsky, V. , Mironova, V. , Scarpella, E. et al. (2024) Analysis of auxin responses in the fern *Ceratopteris richardii* identifies the developmental phase as a major determinant for response properties. Development, 151, dev203026. 39324436 10.1242/dev.203026 PMC11449451

[tpj70543-bib-0057] Wu, X. , Liu, X. , Zhang, S. & Zhou, Y. (2023) Cell division and meristem dynamics in fern gametophytes. Plants, 12, 209. 36616337 10.3390/plants12010209 PMC9823664

[tpj70543-bib-0058] Wu, X. , Yan, A. , Liu, X. , Zhang, S. & Zhou, Y. (2022) Quantitative live‐imaging reveals the dynamics of apical cells during gametophyte development in ferns. Quantitative Plant Biology, 3, e25. 37077984 10.1017/qpb.2022.21 PMC10095955

[tpj70543-bib-0059] Wu, X. , Yan, A. , Mcadam, S.M. , Banks, J.A. , Zhang, S. & Zhou, Y. (2021) Timing of meristem initiation and maintenance determines the morphology of fern gametophytes. Journal of Experimental Botany, 72, 6990–7001. 34181730 10.1093/jxb/erab307

[tpj70543-bib-0060] Wu, X. , Yan, A. , Yang, X. , Banks, J.A. , Zhang, S. & Zhou, Y. (2022) Cell growth dynamics in two types of apical meristems in fern gametophytes. The Plant Journal, 111, 149–163. 35451138 10.1111/tpj.15784 PMC9541313

[tpj70543-bib-0061] Wu, X. , Yan, A. , Yang, X. , McAdam, S.A.M. , Liu, X. , Zhang, S. et al. (2025) Dynamic cell division and growth during de novo meristem formation in epiphytic Fern gametophytes. Journal of Experimental Botany, 76, eraf206. 10.1093/jxb/eraf206 40357922

[tpj70543-bib-0062] Xie, C. , Zhang, C. , Liu, X. & Zhou, Y. (2025) The cellular basis of meristem development in fern gametophytes. Biochemical Society Transactions, 53, BST20240728. 39945720 10.1042/BST20240728 PMC12224904

[tpj70543-bib-0063] Yamane, H. , Nohara, K. , Takahashi, N. , Corey, E.J. , Myers, A.G. , Schraudolft, H. et al. (1987) Biological activity of antheridic acid, an antheridiogen of *Anemia phyllitidis* . Phytochemistry, 26, 1855–1857.

[tpj70543-bib-0064] Yamane, H. , Takahashi, N. , Takeno, K. & Furuya, M. (1979) Identification of gibberellin a 9 methyl ester as a natural substance regulating formation of reproductive organs in *Lygodium japonicum* . Planta, 147, 251–256. 24311041 10.1007/BF00388747

[tpj70543-bib-0065] Yang, X. , Yan, A. , Liu, X. , Volkening, A. & Zhou, Y. (2025) Single cell‐derived multicellular meristem: insights into male‐to‐hermaphrodite conversion and de novo meristem formation in Ceratopteris. Development, 152, DEV204411. 39817858 10.1242/dev.204411 PMC11883269

[tpj70543-bib-0066] Yoshida, T. , Fujita, Y. , Sayama, H. , Kidokoro, S. , Maruyama, K. , Mizoi, J. et al. (2010) AREB1, AREB2, and ABF3 are master transcription factors that cooperatively regulate ABRE‐dependent ABA signaling involved in drought stress tolerance and require ABA for full activation. The Plant Journal, 61, 672–685. 19947981 10.1111/j.1365-313X.2009.04092.x

[tpj70543-bib-0067] Youngstrom, C.E. , Geadelmann, L.F. , Irish, E.E. & Cheng, C.L. (2019) A fern WUSCHEL‐RELATED HOMEOBOX gene functions in both gametophyte and sporophyte generations. BMC Plant Biology, 19, 1–13. 31601197 10.1186/s12870-019-1991-8 PMC6788082

