# Supplementary material for: Cellular basis of ABA‐induced de novo meristem formation and sex‐type conversion in Ceratopteris gametophytes
Source: Plant J. 2025 Nov 4;124(3):e70543. doi: 10.1111/tpj.70543 (PMC12583953; doi:10.1111/tpj.70543)
Supplement: Supplementary file 1 — Figure S1. Live imaging of hermaphrodites and males in the absence of antheridiogen with mock treatment. Figure S2. Confocal imaging of a Ceratopteris male gametophyte expressing the pCrUBQ10::H2B‐GFP::3'CrUBQ10 nuclear marker. Figure S3. Confocal imaging of a Ceratopteris hermaphrodite gametophyte expressing the pCrUBQ10::H2B‐GFP::3'CrUBQ10 nuclear marker. Figure S4. Time‐lapse confocal imaging of a male gametophyte from 0 to 90 h in the presence of both antheridiogen and ABA. Figure S5. Time‐lapse confocal imaging of the first male gametophyte (Sample 3) from 96 to 186 h reveals de novo meristem development in the presence of both antheridiogen and ABA. Figure S6. Continued time‐lapse imaging of the first male gametophyte (Sample 3) from 192 to 276 h shows meristem notch formation in the presence of both antheridiogen and ABA. Figure S7. Time‐lapse confocal imaging of the second male gametophyte (Sample 5) from 0 to 90 h in the presence of both antheridiogen and ABA. Figure S8. Time‐lapse confocal imaging of the second male gametophyte (Sample 5) from 96 to 186 h reveals de novo meristem development in the presence of both antheridiogen and ABA. Figure S9. Continued time‐lapse imaging of the second male gametophyte (Sample 5) shows meristem notch formation in the presence of both antheridiogen and ABA. Figure S10. Time‐lapse confocal imaging of the third male gametophyte (Sample 17) from 0 to 90 h in the presence of both antheridiogen and ABA. Figure S11. Time‐lapse confocal imaging of the third male gametophyte (Sample 17) from 96 to 186 h reveals de novo meristem development in the presence of both antheridiogen and ABA. Figure S12. Continued time‐lapse imaging of the third male gametophyte (Sample 17) shows meristem notch formation in the presence of both antheridiogen and ABA. Figure S13. Time‐lapse confocal imaging of a male gametophyte from 0 to 90 h in the presence of antheridiogen alone. Figure S14. Continued time‐lapse imaging of the first mock sample (Mo [file TPJ-124-0-s004.pdf]

Supplementary Information

Supplementary Figures

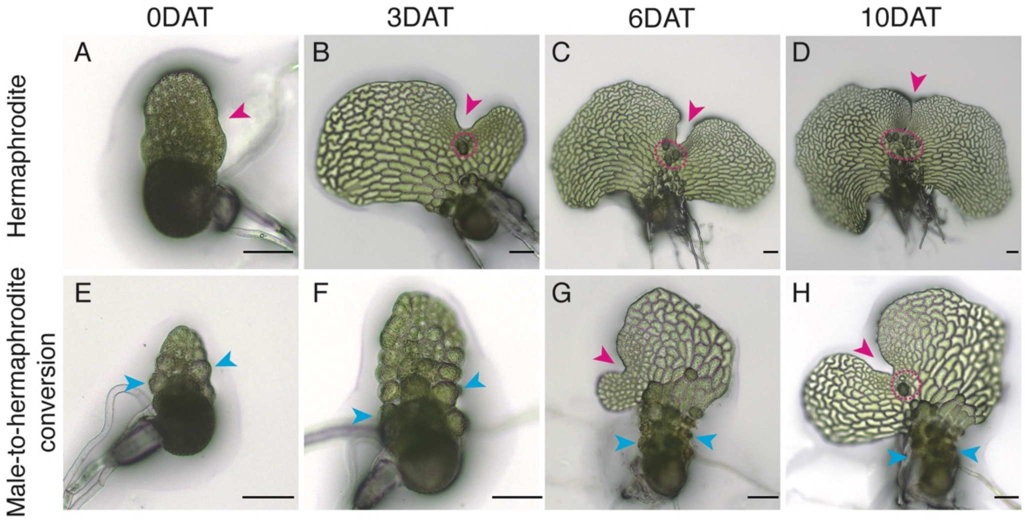

Figure S1. Live imaging of hermaphrodites and males in the absence of antheridiogen with mock treatment. (A-D) A representative hermaphroditic gametophyte grown on FM (without antheridiogen) with mock treatment, developing the meristem and adjacent archegonia. (E-H) A representative male gametophyte grown on FM (without antheridiogen) with mock treatment, converting into a hermaphrodite and forming the *de novo* meristem and archegonia. (A-H) At 2 DAG (A, E), gametophytes were transferred onto fresh FM containing mock, and time-lapse light micrographs were taken at the indicated days after treatment (DAT). Magenta arrowheads indicate the initiating meristem in (A) and the meristem notches in (B-D, G-H). Magenta dashed circles indicate archegonia. Blue arrowheads indicate representative antheridia. Scale bars: 100 µm. Samples shown in Figure S1 and Figure 1 were cultured under the same growth conditions, allowing direct comparison of gametophytes in response to different treatments.

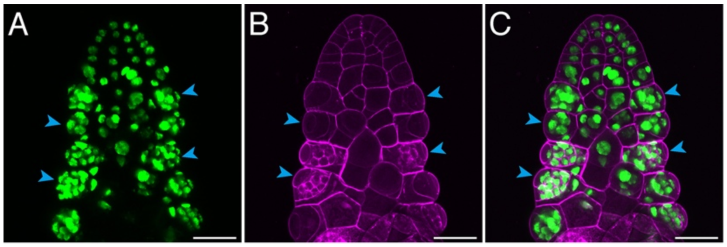

Figure S2. Confocal imaging of a Ceratopteris male gametophyte expressing the *pCrUBQ10::H2B-GFP::3'CrUBQ10* nuclear marker. Z-projection views of a representative male (4 DAG) imaged by laser scanning confocal microscopy. Blue arrowheads (A-C) highlight representative antheridia. (A) GFP (green); (B) PI counterstain (magenta, showing cell outlines); (C) Merged channels of GFP and PI. Scale bars: 50 μm.

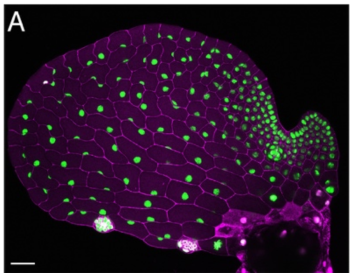

Figure S3. Confocal imaging of a Ceratopteris hermaphrodite gametophyte expressing the *pCrUBQ10::H2B-GFP::3'CrUBQ10* nuclear marker. Z-projection view of the hermaphrodite (4 DAG) imaged by laser scanning confocal microscopy. (A) Merged GFP (green) and PI (magenta). Scale bar: 50 µm.

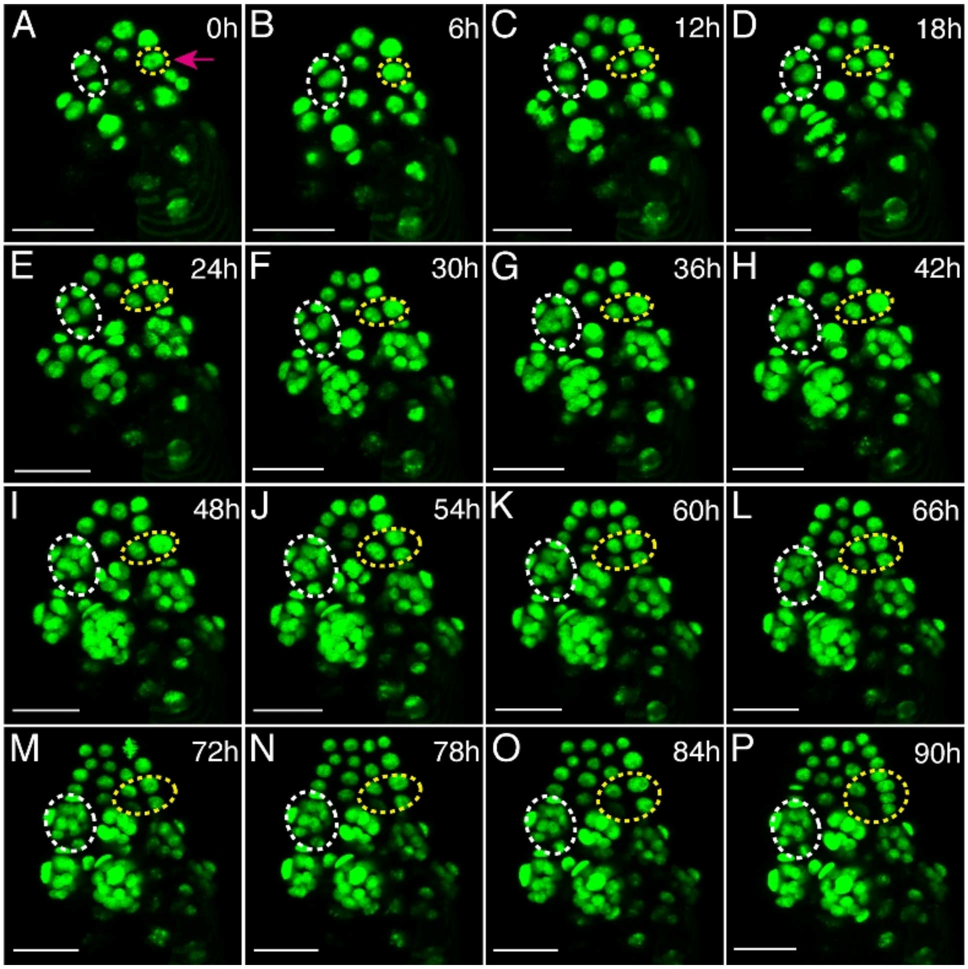

Figure S4. Time-lapse confocal imaging of a male gametophyte from 0-90 h in the presence of both antheridiogen and ABA. (A-P) Z-projection views of a male gametophyte (Sample 3) expressing the *pCrUBQ10::H2B-GFP::3'CrUBQ10* transgenic reporter. (A) At 2 DAG, the male gametophyte was transferred from CFM to CFM with 2.5 µM ABA and imaged at 0 h by laser scanning confocal microscopy. (A-P) Live imaging was conducted every six hours from 0 h to 90 h (A-P). A magenta arrow (A) marks the site of initial cell proliferation associated with *de novo* meristem formation. Yellow dashed circles indicate the lineage of the meristem progenitor cell (MPC) across time points. White dashed circles highlight one representative antheridium. GFP signal is shown in green. Scale bars: 50 µm. At least three biological replicates were live-imaged under identical conditions at 6-hour intervals, all showing comparable results. The complete confocal image series for this sample over the first 90 h is shown in this figure, and the complete series covering the subsequent 96-276 h is provided in Figs. S5 and S6. Images of several representative time points from this sample are also presented in Fig. 2 (Fig. 2A-F correspond to

Fig. S4A, D, G, J, M, and P). Full time series for the other two samples are presented in Figs. S7-S9 and S10-S12, respectively.

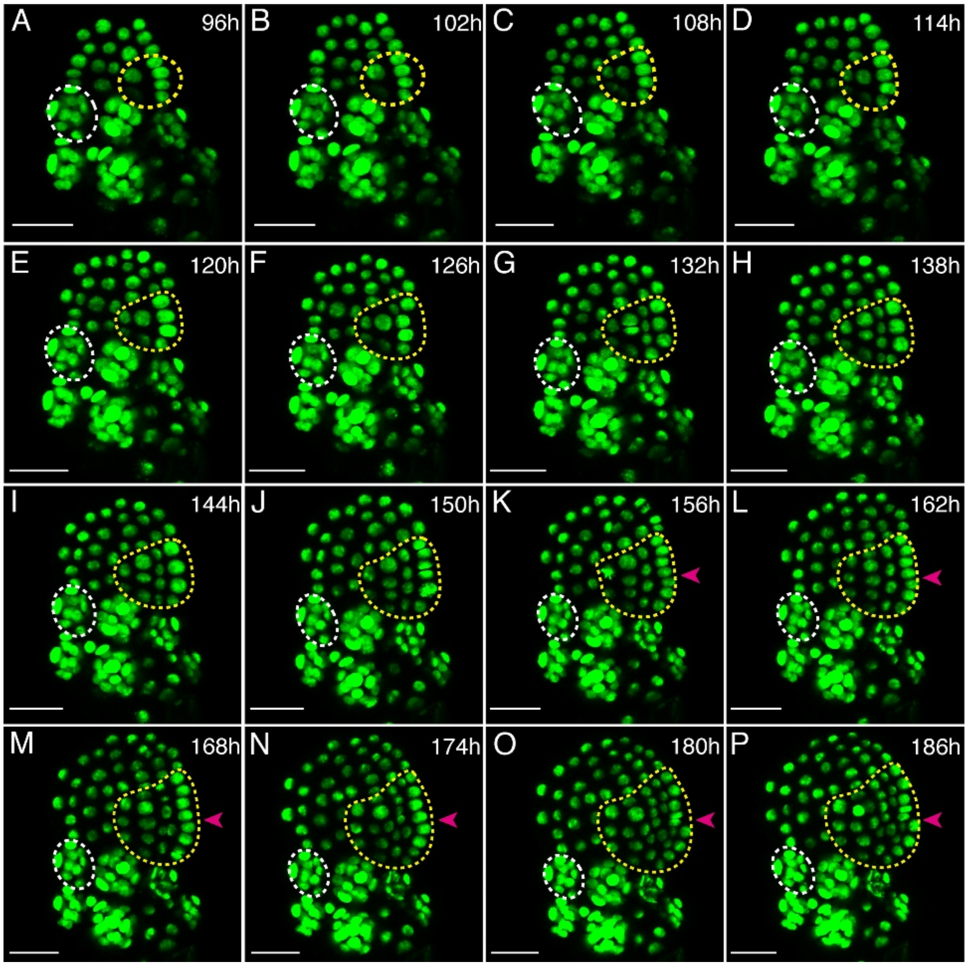

Figure S5. Time-lapse confocal imaging of the first male gametophyte (Sample 3) from 96-186 h reveals *de novo* meristem development in the presence of both antheridiogen and ABA. (A-P) Z-projection views of the same gametophyte shown in Fig. 2 and Fig. S4, expressing the *pCrUBQ10::H2B-GFP::3'CrUBQ10* reporter. Live imaging was continued every six hours from 96 h to 186 h. Magenta arrowheads (K-P) indicate the emergence and formation of a meristem. Yellow dashed circles indicate the MPC lineage during the analyzed time frames. White dashed circles highlight the representative antheridium. GFP signal is shown in green. Scale bars: 50 μm. At least three biological replicates were live-imaged under identical conditions at 6-hour intervals, all showing comparable results. The complete confocal image series for this sample over the 96-186 h is shown in this figure, and the complete series covering the other time frames is provided in Figs. S4 and S6. Images of several representative time points from this sample are also presented in Fig. 2 (Fig. 2G-K correspond to Fig. S5C, F, I, L, O). Full time series for the other two samples are presented in Figs. S7-S9 and S10-S12, respectively.

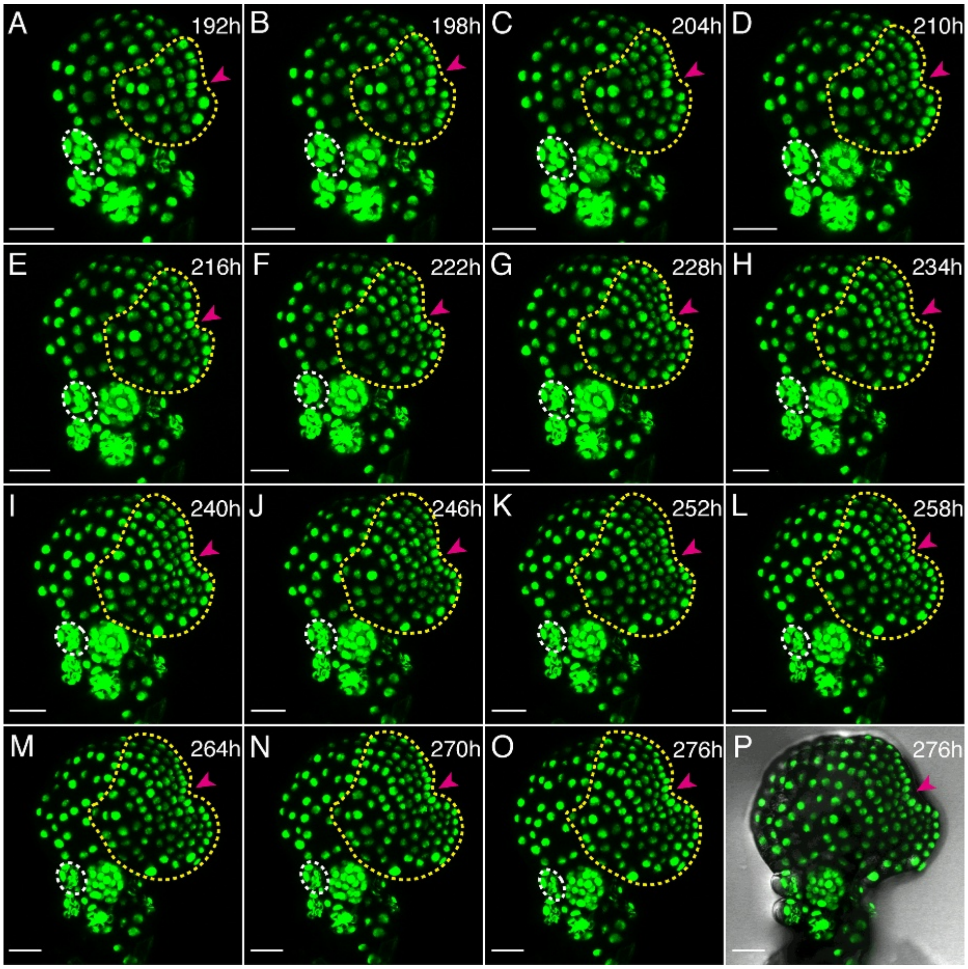

Figure S6. Continued time-lapse imaging of the first male gametophyte (Sample 3) from 192-276 h shows meristem notch formation in the presence of both antheridiogen and ABA. (A-P) Z-projection views of the same male gametophyte shown in Figs. 2 and S4-S5, expressing the *pCrUBQ10::H2B-GFP::3'CrUBQ10* reporter. Live imaging was performed every six hours from 192 h to 276 h. A well-established meristem structure was observed during this period. Magenta arrowheads indicate the concave meristem notch. Yellow dashed circles indicate the MPC lineage during the analyzed time frames. White dashed circles highlight the representative antheridium. (A-O) GFP channel (green) from 192-276 h. (P) a merged view of GFP and DIC channels. Scale bars: 50 µm. At least three biological replicates were live-imaged under identical conditions at 6-hour intervals, all showing comparable results. The complete confocal image series for this sample over the 192-276 h is shown in this figure, and the complete series covering the earlier time frames is provided in Figs. S4 and S5. Images of several representative time points from this sample are

also presented in Fig. 2 (Fig. 2L-R correspond to Fig. S6B, E, H, K, N, O, P). Full time series for the other two samples are presented in Figs. S7-S9 and S10-S12, respectively.

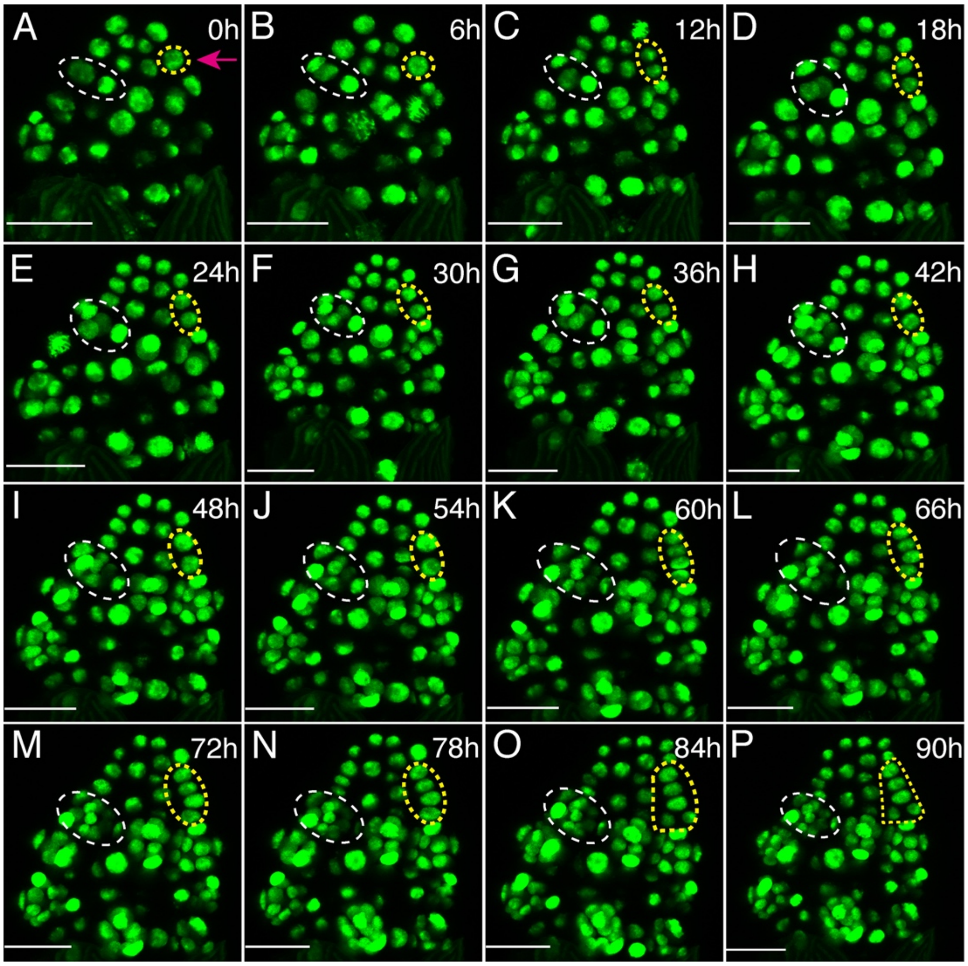

Figure S7. Time-lapse confocal imaging of the second male gametophyte (Sample 5) from 0-90 h in the presence of both antheridiogen and ABA. (A-P) Z-projection views of a male gametophyte (Sample 5) expressing the *pCrUBQ10::H2B-GFP::3'CrUBQ10* transgenic reporter. (A) At 2 DAG, the male gametophyte was transferred from CFM to CFM with 2.5 μM ABA and imaged at 0 h by laser scanning confocal microscopy. (A-P) Live imaging was conducted every six hours from 0 h to 90 h (A-P). A magenta arrow (A) marks the site of initial cell proliferation associated with *de novo* meristem formation. Yellow dashed circles indicate the lineage of the meristem progenitor cell (MPC) across time points. White dashed circles highlight one representative antheridium. GFP signal is shown in green. Scale bars: 50 μm. At least three biological replicates were live-imaged under identical conditions at 6-hour intervals, all showing comparable results. The complete confocal image series for this sample over the first 90 h is shown in this figure, and the complete series covering the subsequent 96-270 h is provided in Figs. S8

and S9. Full time series for the other two samples are presented in Figs. S4-S6 and S10-S12, respectively.

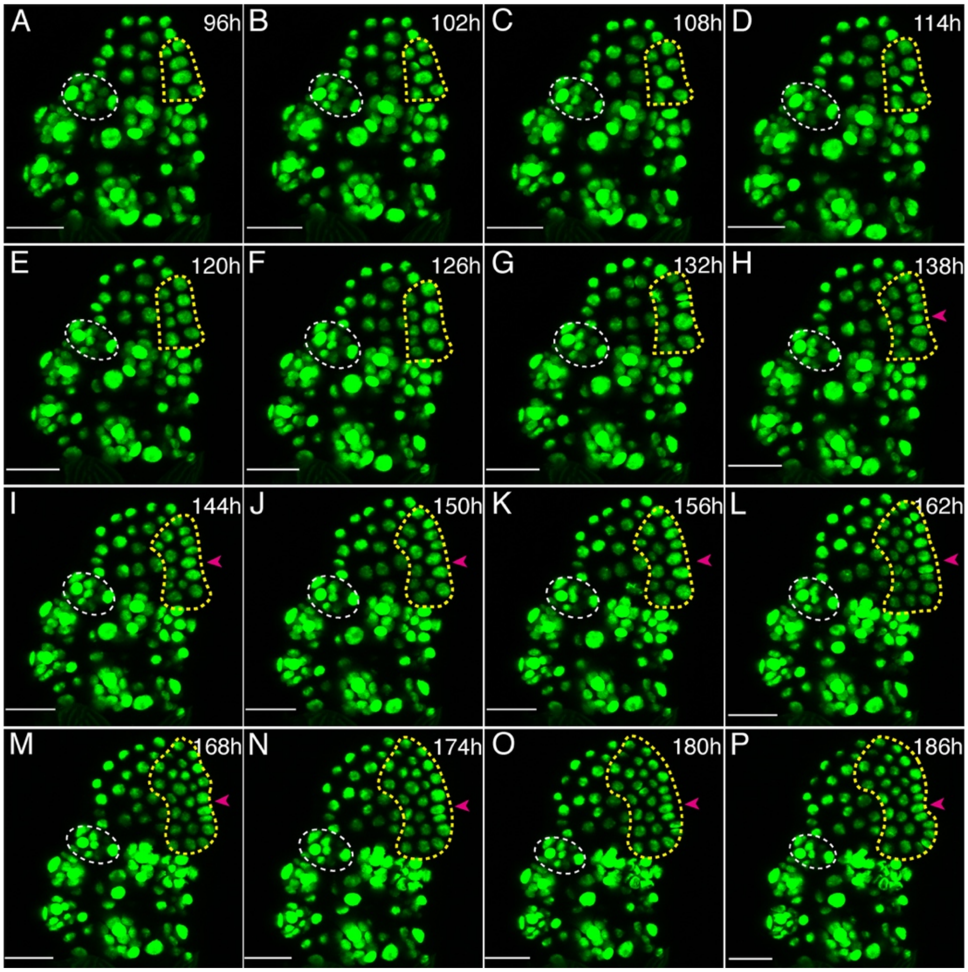

Figure S8. Time-lapse confocal imaging of the second male gametophyte (Sample 5) from 96-186 h reveals *de novo* meristem development in the presence of both antheridiogen and ABA. (A-P) Z-projection views of the same gametophyte (Sample 5) shown in Fig. S7, expressing the *pCrUBQ10::H2B-GFP::3'CrUBQ10* reporter. Live imaging was continued every six hours from 96 h to 186 h. Magenta arrowheads (H-P) indicate the emergence and formation of a meristem. Yellow dashed circles indicate the MPC lineage during the analyzed time frames. White dashed circles highlight the representative antheridium. GFP signal is shown in green. Scale bars: 50 μm. At least three biological replicates were live-imaged under identical conditions at 6-hour intervals, all showing comparable results. The complete confocal image series for this sample over the 96-186 h is shown in this figure, and the complete series covering the other time frames is provided in Figs. S7 and S9. Full time series for the other two samples are presented in Figs. S4-S6 and S10-S12, respectively.

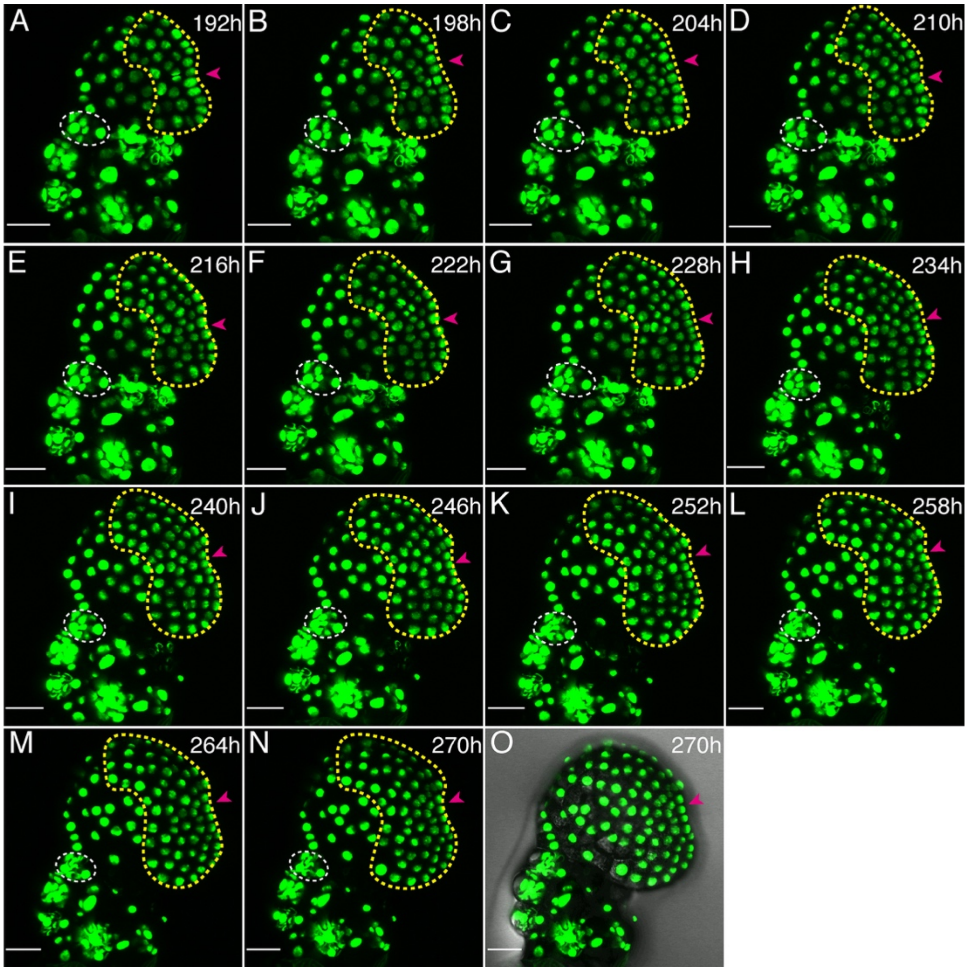

Figure S9. Continued time-lapse imaging of the second male gametophyte (Sample 5) shows meristem notch formation in the presence of both antheridiogen and ABA. (A-O) Z-projection views of the same male gametophyte (Sample 5) shown in Figs. S7-S8, expressing the *pCrUBQ10::H2B-GFP::3'CrUBQ10* reporter. Live imaging was performed every six hours from 192 h to 270 h. A well-established meristem structure was observed during this period. Magenta arrowheads indicate the concave meristem notch. Yellow dashed circles indicate the MPC lineage during the analyzed time frames. White dashed circles highlight the representative antheridium. (A-N) GFP channel (green) from 192-270 h. (O) a merged view of GFP and DIC channels. Scale bars: 50 µm. At least three biological replicates were live-imaged under identical conditions at 6-hour intervals, all showing comparable results. The complete confocal image series for this sample over the 192-270 h is shown in this figure, and the complete series covering the earlier time frames

is provided in Figs. S7 and S8. Full time series for the other two samples are presented in Figs. S4-S6 and S10-S12, respectively.

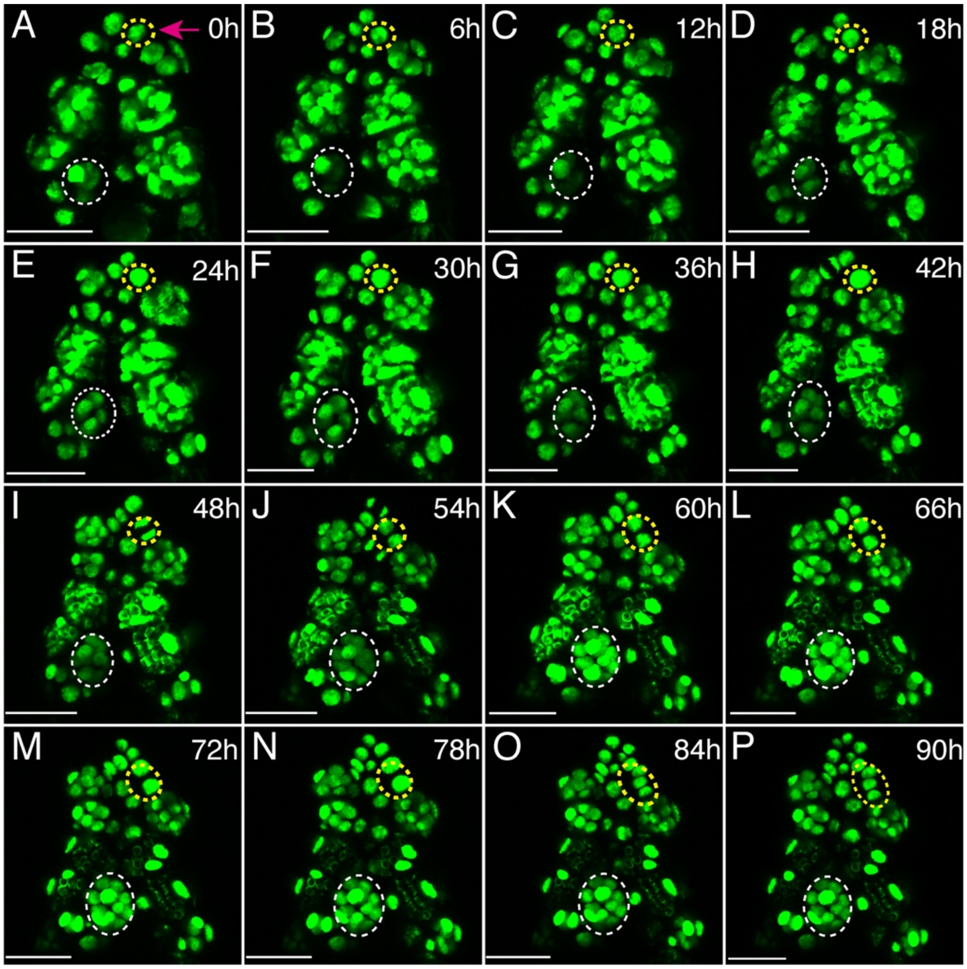

Figure S10. Time-lapse confocal imaging of the third male gametophyte (Sample 17) from 0-90 h in the presence of both antheridiogen and ABA. (A-P) Z-projection views of a male gametophyte (Sample 17) expressing the *pCrUBQ10::H2B-GFP::3'CrUBQ10* transgenic reporter. (A) At 2 DAG, the male gametophyte was transferred from CFM to CFM with 2.5 μM ABA and imaged at 0 h by laser scanning confocal microscopy. (A-P) Live imaging was conducted every six hours from 0 h to 90 h (A-P). A magenta arrow (A) marks the site of initial cell proliferation associated with *de novo* meristem formation. Yellow dashed circles indicate the lineage of the meristem progenitor cell (MPC) across time points. White dashed circles highlight one representative antheridium. GFP signal is shown in green. Scale bars: 50 μm. At least three biological replicates were live-imaged under identical conditions at 6-hour intervals, all showing comparable results. The complete confocal image series for this sample over the first 90 h is shown in this figure, and the complete series covering the subsequent 96-246 h is provided in Figs. S11

and S12. Full time series for the other two samples are presented in Figs. S4-S6 and S7-S9, respectively.

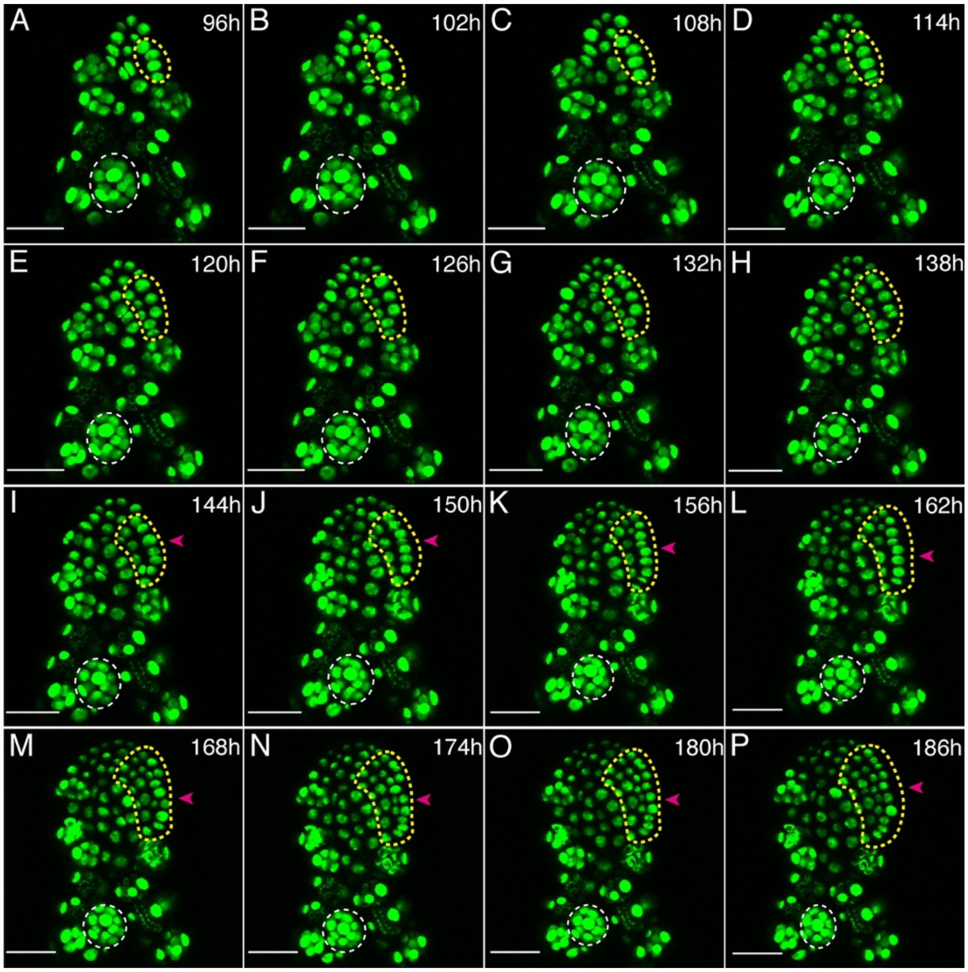

Figure S11. Time-lapse confocal imaging of the third male gametophyte (Sample 17) from 96-186 h reveals *de novo* meristem development in the presence of both antheridiogen and ABA. (A-P) Z-projection views of the same gametophyte (Sample 17) shown in Fig. S10, expressing the *pCrUBQ10::H2B-GFP::3'CrUBQ10* reporter. Live imaging was continued every six hours from 96 h to 186 h. Magenta arrowheads (I-P) indicate the emergence and formation of a meristem. Yellow dashed circles indicate the MPC lineage during the analyzed time frames. White dashed circles highlight the representative antheridium. GFP signal is shown in green. Scale bars: 50 µm. At least three biological replicates were live-imaged under identical conditions at 6-hour intervals, all showing comparable results. The complete confocal image series for this sample over the 96-186 h is shown in this figure, and the complete series covering the other time frames is provided in Figs. S10 and S12. Full time series for the other two samples are presented in Figs. S4-S6 and S7-S9, respectively.

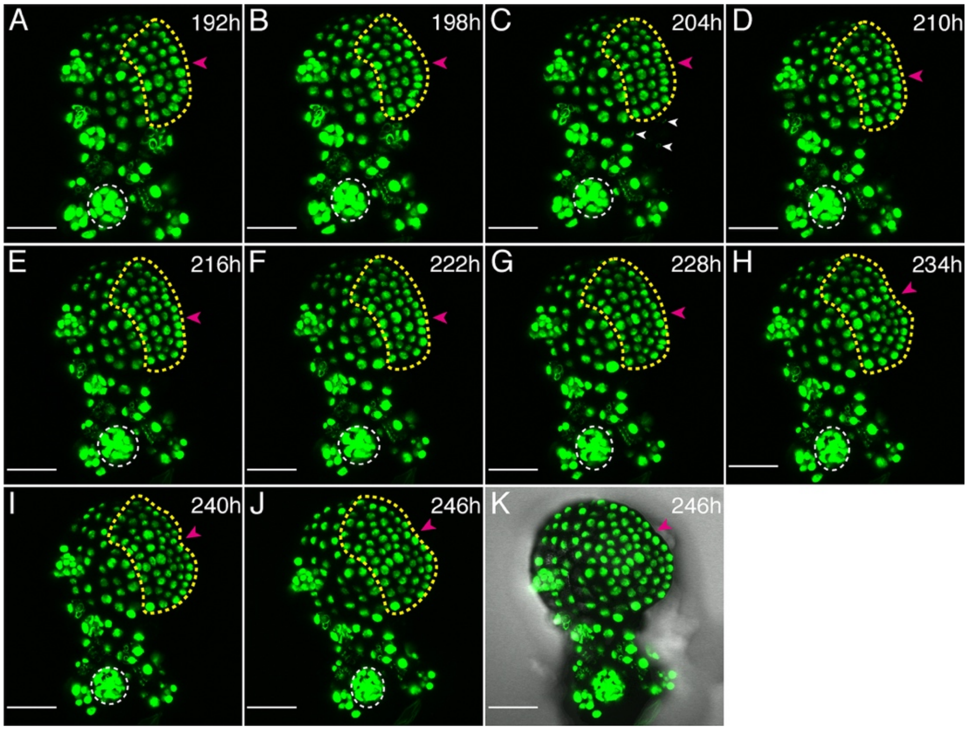

Figure S12. Continued time-lapse imaging of the third male gametophyte (Sample 17) shows meristem notch formation in the presence of both antheridiogen and ABA. (A-K) Z-projection views of the same male gametophyte (Sample 17) shown in Figs. S10-S11, expressing the *pCrUBQ10::H2B-GFP::3'CrUBQ10* reporter. Live imaging was performed every six hours from 192 h to 246 h. A well-established meristem structure was observed during this period. Magenta arrowheads indicate the concave meristem notch. Yellow dashed circles indicate the MPC lineage during the analyzed time frames. White dashed circles highlight the representative antheridium. (A-J) GFP channel (green) from 192-246 h. (K) a merged view of GFP and DIC channels. Scale bars: 50 µm. At least three biological replicates were live-imaged under identical conditions at 6-hour intervals, all showing comparable results. The complete confocal image series for this sample over the 192-246 h is shown in this figure, and the complete series covering the earlier time frames is provided in Figs. S10 and S11. Full time series for the other two samples are presented in Figs. S4-S6 and S7-S9, respectively.

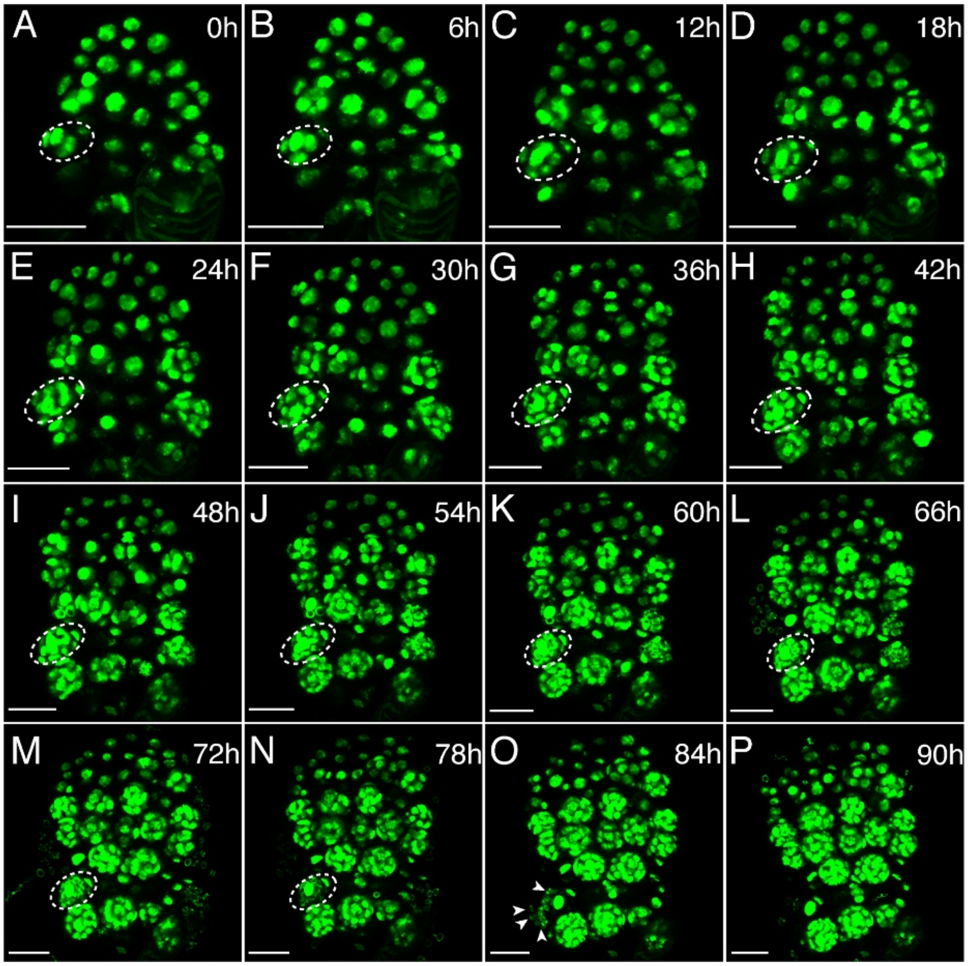

Figure S13. Time-lapse confocal imaging of a male gametophyte from 0-90 h in the presence of antheridiogen alone. (A-P) Z-projection views of a male gametophyte (Mock Sample 5) expressing the *pCrUBQ10::H2B-GFP::3'CrUBQ10* reporter. (A) At 2 DAG, the male gametophyte was transferred from CFM to CFM with mock treatment and imaged at 0 h by laser scanning confocal microscopy. (A-P) Live imaging was performed every six hours from 0 h to 90 h. White dashed circles highlight one representative antheridium. White arrowheads in (O) indicate motile sperm released from the representative mature antheridium. GFP signal is shown in green. Scale bars: 50 µm. At least three biological replicates were live-imaged under identical conditions at 6-hour intervals, all showing comparable results. The complete confocal image series for this sample over the first 90 h is shown in this figure, and the complete series covering the subsequent time frame is provided in Fig. S14. Images of several representative time points from this sample are also presented in Fig. 3 (Fig. 3A-F correspond to Fig. S13A, D, G, J, M, P). Full time series for the other two samples are presented in Figs. S15-S16 and S17-S18, respectively.

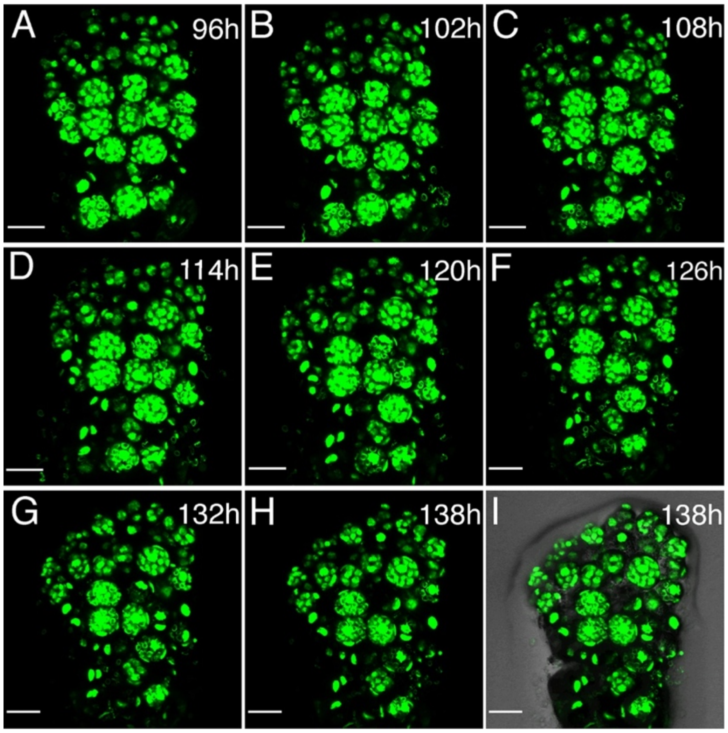

Figure S14. Continued time-lapse imaging of the first mock sample (Mock Sample 5) from 96-138 h reveals antheridium maturation in the presence of antheridiogen alone. (A-Y) Z-projection views of the same male gametophyte (Mock Sample 5) shown in Fig. 3 and Fig. S13, expressing the *pCrUBQ10::H2B-GFP::3'CrUBQ10* reporter. Live imaging was performed every six hours from 96 h to 138 h, during which multiple antheridia matured and ruptured. (A-H) GFP channel (green) from 96-138 h. (I) a merged view of GFP and DIC channels. Scale bars: 50 µm. At least three biological replicates were live-imaged under identical conditions at 6-hour intervals, all showing comparable results. The complete confocal image series for this sample over the 96-138 h is shown in this figure, and the complete series covering the earlier time frame is provided in Fig. S13. Images of several representative time points from this sample are also presented in Fig. 3 (Fig. 3G-J correspond to Fig. S14C, F, H, I). Full time series for the other two samples are presented in Figs. S15-S16 and S17-S18, respectively.

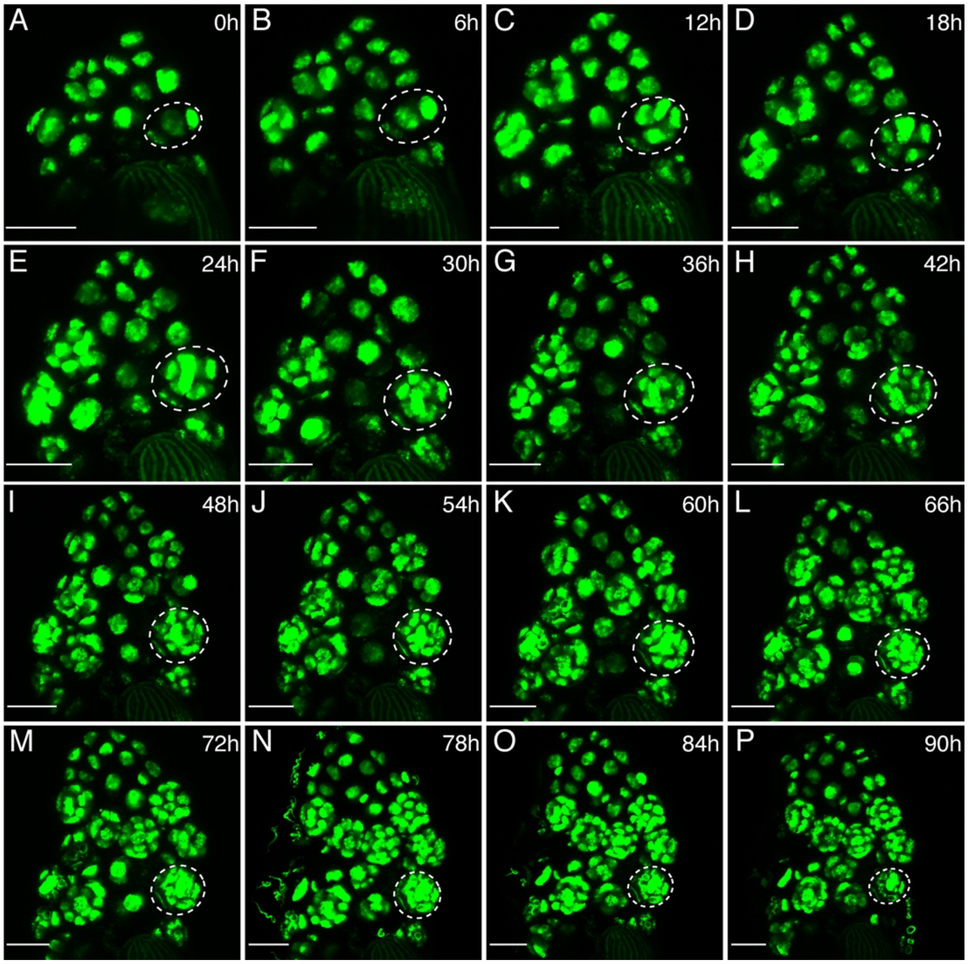

Figure S15. Time-lapse confocal imaging of the second male gametophyte (Mock Sample 1) from 0-90 h in the presence of antheridiogen alone. (A-P) Z-projection views of a male gametophyte (Mock Sample 1) expressing the *pCrUBQ10::H2B-GFP::3'CrUBQ10* reporter. (A) At 2 DAG, the male gametophyte was transferred from CFM to CFM with mock treatment and imaged at 0 h by laser scanning confocal microscopy. (A-P) Live imaging was performed every six hours from 0 h to 90 h. White dashed circles highlight one representative antheridium. GFP signal is shown in green. Scale bars: 50 µm. At least three biological replicates were live-imaged under identical conditions at 6-hour intervals, all showing comparable results. The complete confocal image series for this sample over the first 90 h is shown in this figure, and the complete series covering the subsequent time frame is provided in Fig. S16. Full time series for the other two samples are presented in Figs. S13-S14 and S17-S18, respectively.

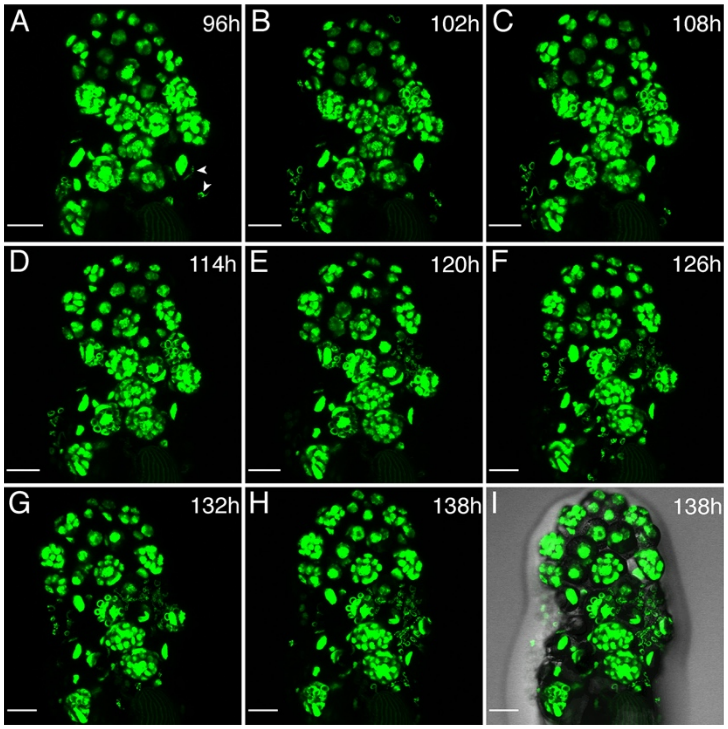

Figure S16. Continued time-lapse imaging of the second sample (Mock Sample 1) from 96-138 h reveals antheridium maturation in the presence of antheridiogen alone. (A-H) Z-projection views of the same male gametophyte (Mock Sample 1) shown in Fig. S15, expressing the *pCrUBQ10::H2B-GFP::3'CrUBQ10* reporter. Live imaging was performed every six hours from 96 h to 138 h, during which multiple antheridia matured and ruptured. Arrows in (A) indicate motile sperm released from mature antheridia. (A-H) GFP channel (green) from 96-138 h. (I) a merged view of GFP and DIC channels. Scale bars: 50 µm. At least three biological replicates were live-imaged under identical conditions at 6-hour intervals, all showing comparable results. The complete confocal image series for this sample over the 96-138 h is shown in this figure, and the complete series covering the earlier time frame is provided in Fig. S15. Full time series for the other two samples are presented in Figs. S13-S14 and S17-S18, respectively.

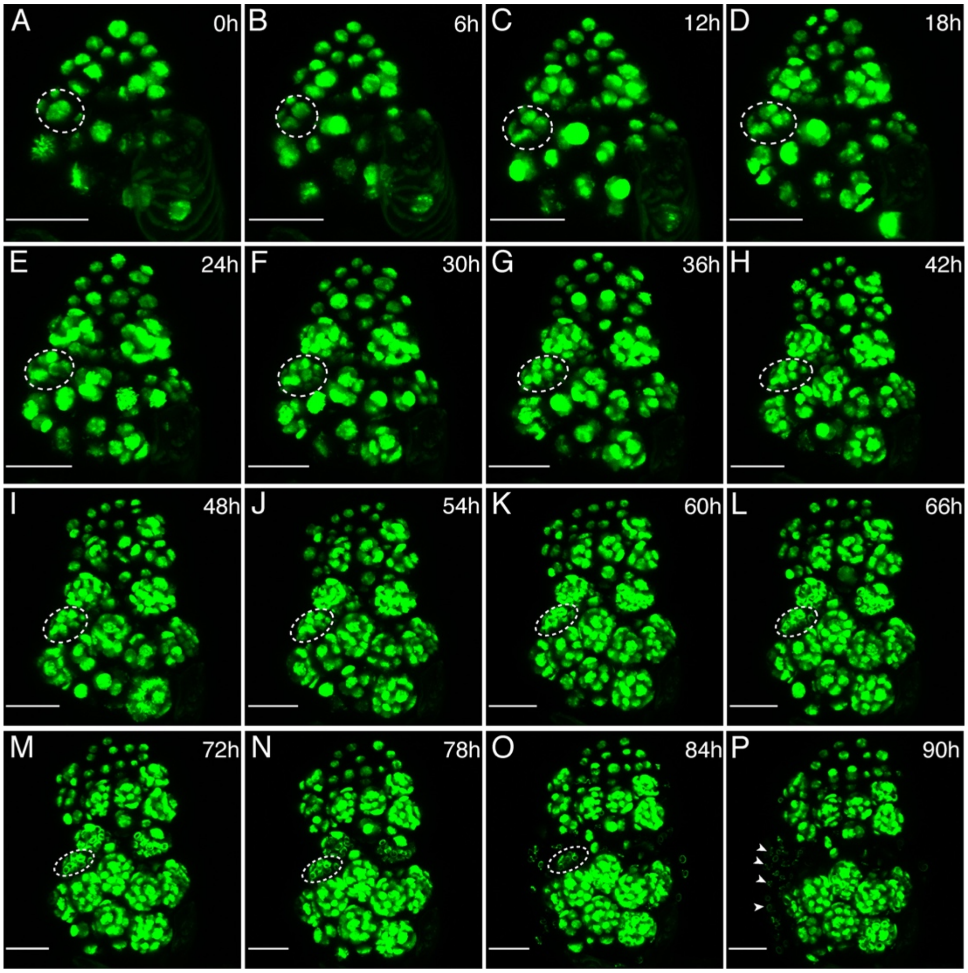

Figure S17. Time-lapse confocal imaging of the third male gametophyte (Mock Sample 2) from 0-90 h in the presence of antheridiogen alone. (A-P) Z-projection views of a male gametophyte (Mock Sample 2) expressing the *pCrUBQ10::H2B-GFP::3'CrUBQ10* reporter. (A) At 2 DAG, the male gametophyte was transferred from CFM to CFM with mock treatment and imaged at 0 h by laser scanning confocal microscopy. (A-P) Live imaging was performed every six hours from 0 h to 90 h. White dashed circles highlight one representative antheridium. Arrows in (P) indicate motile sperm released from mature antheridia. GFP signal is shown in green. Scale bars: 50 µm. At least three biological replicates were live-imaged under identical conditions at 6-hour intervals, all showing comparable results. The complete confocal image series for this sample over the first 90 h is shown in this figure, and the complete series covering the subsequent time frame is provided in Fig. S18. Full time series for the other two samples are presented in Figs. S13-S14 and S15-S16, respectively.

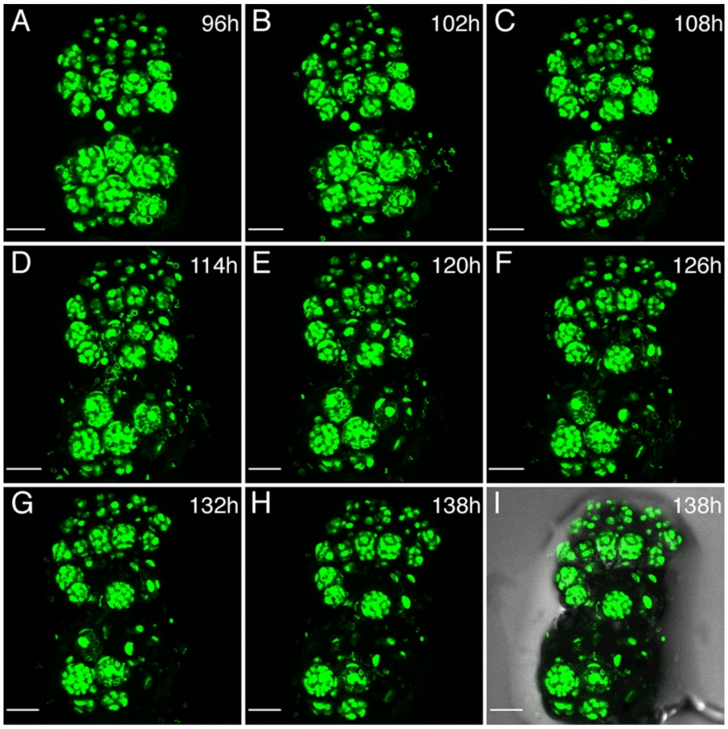

Figure S18. Continued time-lapse imaging of the third sample (Mock Sample 2) from 96-138 h reveals antheridium maturation in the presence of antheridiogen alone. (A-H) Z-projection views of the same male gametophyte (Mock Sample 2) shown in Fig. S17, expressing the *pCrUBQ10::H2B-GFP::3'CrUBQ10* reporter. Live imaging was performed every six hours from 96 h to 138 h, during which multiple antheridia matured and ruptured. (A-H) GFP channel (green) from 96-138 h. (I) a merged view of GFP and DIC channels. Scale bars: 50 µm. At least three biological replicates were live-imaged under identical conditions at 6-hour intervals, all showing comparable results. The complete confocal image series for this sample over the 96-138 h is shown in this figure, and the complete series covering the earlier time frame is provided in Fig. S17. Full time series for the other two samples are presented in Figs. S13-S14 and S15-S16, respectively.

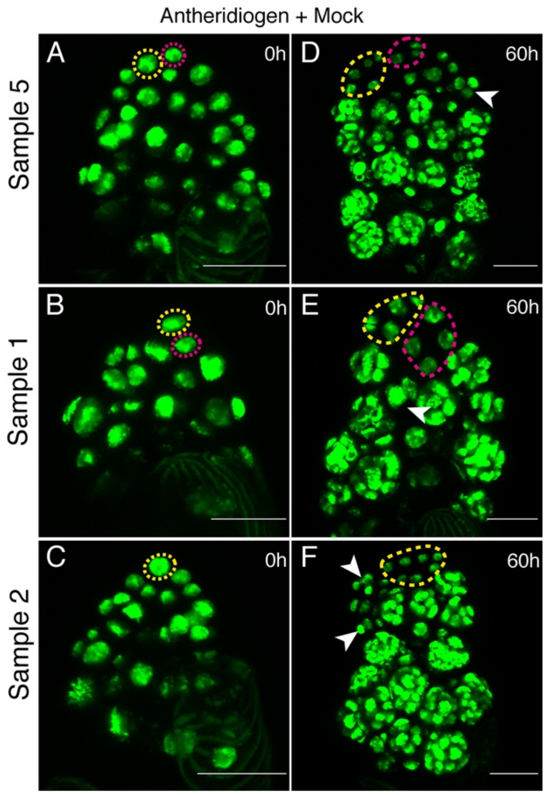

Figure S19. Non-antheridium cell lineages from three male gametophytes in the presence of antheridiogen alone. (A-F) Non-antheridium lineages (outlined in yellow and magenta dashed lines) in three independent male samples at 0 h (A-C) and 60 h (D-F) of live imaging. Panels correspond to zoomed-in views from other figures: A, Fig. 3A and Fig. S13A; D, Fig. S13K; B, Fig. S15A; E, Fig. S15K; C, Fig. S17A; F, Fig. S17K. Yellow dashed outlines indicate the largest non-antheridium lineage, and magenta dashed outlines indicate the second-largest non-antheridium lineage. White arrowheads indicate representative developing antheridia. Scale bars: 50 µm. Quantitative cell counts for the highlighted lineages (yellow and magenta) from the three samples at the indicated time points are included in Supplementary Tables S4-S6, respectively.

Supplementary Tables S1-S7

Table S1. Source Data for Figure 4G. Total cell counts of the MPC lineage (yellow) and the second-largest non-antheridium lineage (magenta) in Sample 3, at various time points in the presence of both antheridiogen and ABA.

Table S2. Source Data for Figure 4H. Total cell counts of the MPC lineage (yellow) and the second-largest non-antheridium lineage (magenta) in Sample 5, at various time points in the presence of both antheridiogen and ABA.

Table S3. Source Data for Figure 4I. Total cell counts of the MPC lineage (yellow) and the second-largest non-antheridium lineage (magenta) in Sample 17, at various time points in the presence of both antheridiogen and ABA.

Table S4. Total cell counts of the non-antheridium lineages (yellow and magenta) in mock-treated Sample 5, at various time points (0-60 h) in the presence of antheridiogen alone.

Table S5. Total cell counts of the non-antheridium lineages (yellow and magenta) in mock-treated Sample 1, at various time points (0-60 h) in the presence of antheridiogen alone.

Table S6. Total cell counts of the non-antheridium lineage (yellow) in mock-treated Sample 2, at various time points (0-60 h) in the presence of antheridiogen alone.

Table S7. Quantitative comparison of cell division events in the MPC lineage (yellow) during male-to-hermaphrodite conversion, triggered either by antheridiogen removal or by ABA treatment.
